# Use of digital health in task-sharing for prevention and management of non-communicable diseases in Africa: A scoping review

**DOI:** 10.1371/journal.pgph.0006746

**Published:** 2026-07-24

**Authors:** Nongiwe Linette Mhlanga, Sikhumbuzo A. Mabunda, Rohina Joshi, Azeb Gebresilassie Tesema

**Affiliations:** 1 School of Public Health, Walter Sisulu University, Mthatha, South Africa; 2 Global Centre for Human Resources for Health Intelligence, Walter Sisulu University, Mthatha, South Africa; 3 School of Population Health, University of New South Wales (UNSW), Sydney, Australia; 4 The George Institute for Global Health, University of New South Wales (UNSW), Sydney, Australia; Universiti Malaya, MALAYSIA

## Abstract

Task-sharing with non-physician health workers (NPHWs), supported by digital health, has emerged as a critical strategy to address workforce shortage and improve the delivery of health services. The use of digital health shows promise in supporting task-sharing for the prevention and management of non-communicable diseases (NCDs) elsewhere; however, the evidence from Africa remains limited. This study aims to review the use of digital health in task-sharing for the prevention and management of NCDs in Africa. The review described the types of digital health technologies used, their functions, effectiveness, and what affects optimal use. The Joanna Briggs Institute (JBI) protocol for scoping reviews was employed, and searches were conducted across three databases—PubMed, Scopus, and Google Scholar—for studies published up to August 2025. The search identified 4,857 citations, of which 71 full texts were screened. Fifteen studies from eight African countries were included in the review, with the largest proportion (26.7%; 4/15) originating from South Africa. mHealth was the most frequently used digital health technology in 46.7% (7/15) of studies, with community health workers as the primary users. The review found that NPHWs utilized digital health technologies for prevention, screening, treatment delivery, and supervision. Digital health, particularly mHealth, supports NPHWs in task-sharing for NCD prevention and management in Africa, despite the limited types of technologies and functions. The use of digital health was limited by low digital literacy, poor internet connectivity, and inadequate electricity supply. Effective implementation requires governance, sustainable funding, infrastructure, workforce development, and robust data systems tailored to the specific contexts of African countries. PROTOCOL REGISTRATION NUMBER: https://doi.org/10.17605/OSF.IO/Q2XF9

## Introduction

In Africa, there has been an increase in non-communicable diseases (NCDs), such as cardiovascular conditions, cancers, diabetes, and chronic respiratory conditions, for the past two decades, and by 2019, NCDs accounted for 2.1 million premature deaths [1–3]. The burden is projected to grow even further in the next decade, with an additional 28 million NCD-related deaths [4], intensifying pressure on already strained health systems [2,3]. Non-communicable diseases, also known as chronic conditions, include cardiovascular conditions, cancers, diabetes, and chronic respiratory conditions, as well as other conditions, including mental health conditions [5,6]. To address the growing burden, African countries have committed to implementing the Global Action Plan for the Prevention and Control of NCDs 2013–2030, which emphasizes strengthening health systems through people-centered primary health care and advancing Universal Health Coverage [7].

Despite progress in expanding primary health care for NCDs, significant gaps persist across many African countries’ health systems. These include underinvestment in health, critical shortages of trained health workforce, and inadequate integration of NCD services into routine care. The average health workforce density in the region remains critically low, at 1.55 per 1,000 people—compared to 4.45 per 1,000 required by the World Health Organization (WHO) to achieve Universal Health Coverage [8]. Addressing persistent health worker shortages, uneven distribution, and gaps in training is essential to delivering quality and equitable NCD care and strengthening the health system.

Task-sharing has been recognized as an innovative and effective approach for addressing health workforce needs [7,9]. Task-sharing refers to a strategy of rationally and optimally distributing responsibilities, ideally to less specialized health workers, to address high service demand in the face of a shortage of skilled providers, thereby making efficient use of the available workforce [10]. Evidence suggests that non-physician health workers (NPHWs)—such as community health workers (CHWs)—can deliver basic health services that have typically been delivered by professional health workers [11,12]. The success of task-sharing is largely determined by supportive regulatory frameworks, adequate supervision, standardized training, the availability of resources, and technologies that augment NPHWs’ capabilities [13].

Digital health is increasingly playing a supportive role in facilitating task-sharing for health services [14]. Digital health refers to the appropriate use of technologies to improve population and individual health, as well as improve patient care by using artificial intelligence (AI) to process clinical and genetic data [15]. The WHO Strategy on Digital Health 2020-2025 fosters digital health use through advocating for digitally enabled health systems [16], with evidence from Low-to-Middle Income Countries (LMICs) suggesting that digital health has been used to deliver NCD services, such as mental health services [14,17].

While the use of digital health demonstrates promise in supporting task-sharing for preventing and managing NCDs elsewhere [18], evidence from Africa —particularly on the use of digital health technologies and the factors that influence their optimal implementation—remains limited. Therefore, this study aims to review the use of digital health in task-sharing to prevent and manage NCDs in Africa. The review also aims to identify the characteristics of health workers who use digital technologies, the contexts in which digital health is used, and the effectiveness of digital health technologies in supporting task-sharing approaches for NCD prevention and management.

## Materials and methods

The review was conducted following the Joanna Briggs Institute (JBI) methodology, which builds upon the scoping review framework originally proposed by Arksey and O’Malley (2005) [19,20]. The study was reported using the Preferred Reporting Items for Systematic Reviews and Meta-Analysis for Scoping Reviews (PRISMA-ScR) [21], included as S1 PRISMA-ScR Checklist. This scoping review protocol was registered before study initiation on the Open Science Framework, registration number https://doi.org/10.17605/OSF.IO/Q2XF9

The study was guided by the research question: How is digital health used by African NPHWs for task-sharing in NCD prevention and management? Digital health refers to the appropriate use of technologies to improve population and individual health, as well as improve patient care by using technologies to process clinical and genetic data [15]. “Digital health technologies” is a broad umbrella term that encompasses all technologies used to improve health and healthcare delivery, including electronic health records, web-based applications, blockchain technology, and mHealth [14,16]. mHealth refers to the use of mobile communications like smart mobile phones, telephones, and smartphone applications for health services and information [22]. Therefore, we outlined the technologies and their functions in NCD prevention and management.

The review also aimed to answer additional questions: what are the characteristics of NPHWs using digital health for task-sharing in NCD prevention and management, in what contexts do the NPHWs use digital health for task-sharing in NCD prevention and management, is the use of digital health in task-sharing effective, and what affects optimal use of digital health?

To identify relevant sources, three databases—PubMed, Scopus, and Google Scholar (first 25 pages, with 10 articles per page)—were searched using the key search terms “digital health, “non-communicable diseases”, and “task-sharing”. A librarian was consulted to develop the search strategy and facilitate the retrieval of articles for full-text screening. The database search strategy was developed on the 7^th^ of April 2025, and the search was conducted until the 3^rd^ of August 2025. The full database search strategy is shown in S1 Appendix. The reference lists of identified studies were also searched.

The review included studies published from 2014 to August 2025, allowing for an analysis spanning more than 10 years of changes in digital health technology. All types of study designs were included, and only studies published in English were included to minimise translation bias. Studies that did not specifically identify studies done in Africa were also excluded. Grey literature, such as that found on institutional websites, including those of the WHO and higher education institutions, was excluded because some institutional reports were not based on empirical research. The articles identified for screening were exported to the Mendeley Reference Manager for storage, and Covidence was used to organize the reviewing process. Two reviewers, NLM and NF, independently reviewed the studies against the set eligibility criteria, and this included both title and abstract screening as well as full text screening. Discrepancies between the reviewers were discussed and resolved by consulting the research team, who included AGT, RJ and SAM.

The JBI data extraction tool was modified to guide the data extraction (S2 Appendix) [23]. Extracted data included the author(s), year of publication, study design, title, setting, number of patients included in the studies, and sample characteristics, including the cadre of NPHWs and sample size. The study outcomes related to the review questions were also charted.

A descriptive numerical analysis and content analysis were done to analyze the data. The numerical analysis described the publication years, countries of origin, and study designs of the included studies. Qualitative content analysis using the four steps described by Klenheskel et al. was used to analyze the data [24]. First, units of meaning were identified, consisting of sentences that described the use of digital health. Second, codes were generated to organize sentences with similar meaning under appropriate labels. Third, these codes were grouped into categories, and finally, the categories were synthesized into overarching themes [24].

## Results

**Fig 1:**
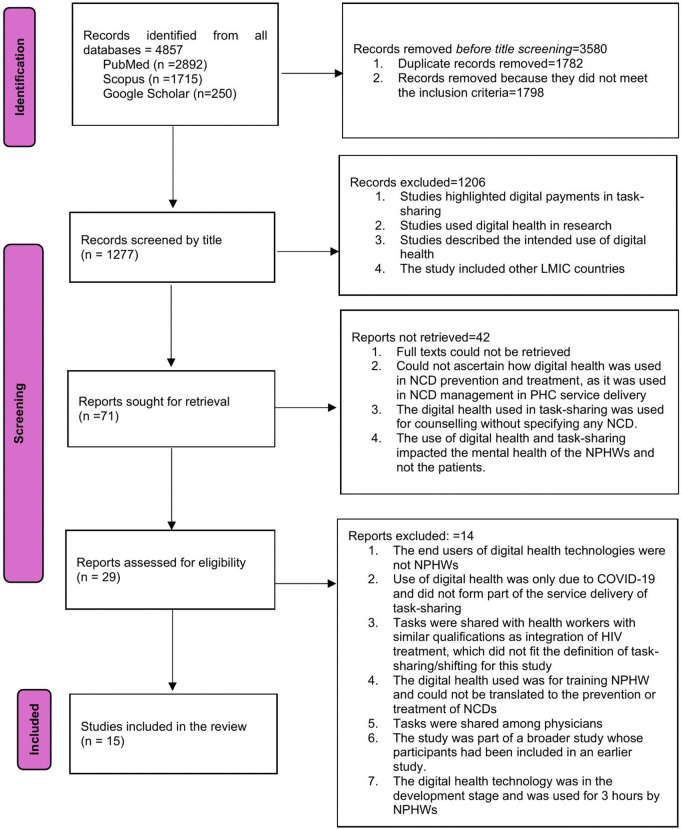
The PRISMA Flow chart.

The search results yielded 4,857 articles; 1,782 duplicates were removed, and 1,798 did not meet the inclusion criteria. A total of 1,277 articles were screened by title. From the studies screened by title only, 1206 were excluded, and 71 were selected for full-text retrieval. A total of 29 were assessed for eligibility. Finally, 15 studies met the inclusion criteria and were included in the study. The decision process using a PRISMA flow diagram adapted from Page et al. [25] illustrates this (Fig 1. The PRISMA Flow chart).

### Characteristics of included studies

This review included 15 studies from eight countries: Ghana, Kenya, Mozambique, Nigeria, Rwanda, South Africa, Tanzania, and Zimbabwe. The largest proportion of studies (26.7%, n = 4) was conducted in South Africa. Regarding the publication year, most (20.0%, n = 3) studies were published in either 2022 or 2023. Most studies (33.3%, n = 5) did not specify the type of settlement where digital health technologies were used. Among the 10 studies that indicated the context of digital health use, most (40.0%; n=4) indicated use in rural areas, while 30.0% (n=3) were in urban settlements. In two studies, digital health was used in both rural and urban areas. Table 1 summarizes the study characteristics and the contexts in which digital health technologies were used.

**Table 1:** Characteristics of selected studies for digital health use for task-sharing for preventing and managing NCDs.

Authors and Year	Study design	Country (Type of settlement)	NCD type	Sample size	NPHW* involved	Type of Digital health technology used	Purpose of digital health in NCD* prevention and management (other functions of digital health)
Doukani et al. (2021) [26]	Cohort study	Kenya (not specified).	Mental Health	52	CHWs*	mHealth (smartphone application: Inuka App).	-mHealth used in screening NCDs* symptoms of common mental health disorders, including depression, using structured questions -mHealth used in providing support for common mental disorders facilitated through decision support.
Chibanda et al. (2016) [27]	Randomised controlled trial.	Zimbabwe (urban).	Mental health	573	Lay health workers	mHealth (telephone calls).	-mHealth is used in the treatment of symptoms of common mental health disorders through facilitating problem-solving skills and following up with patients who missed appointments.
Effah et al. (2022) [28]	Descriptive cross-sectional study.	Ghana (mostly rural with some urban catchment).	Cervical cancer	828	Nurses	mHealth (smartphone imaging and WhatsApp).	-mHealth used in screening cervical cancer through decision support.
Mugisha et al. (2024) [29]	Descriptive qualitative study.	Rwanda (not specified).	Hypertension and diabetes	60	CHWs and Environmental Health Officers	mHealth (smartphone application- Muzima App).	-mHealth was used in screening hypertension and diabetes through screening measurements available on the Muzima App.
Nelissen et al. (2018) [30]	Mixed methods study.	Nigeria (urban).	Hypertension	328	Pharmacists	mHealth (smartphone application).	-mHealth used in the treatment of hypertension through decision support.
Niyibizi et al. (2023) [31]	Action research (analytical cross-sectional).	Rwanda (urban and rural).	Cardiovascular disease	995	CHWs	mHealth (Smartphone application: KoboCollect App).	-mHealth used in screening cardiovascular disease risks through a BMI*-based algorithm on the KoboCollect App.
O’Grady et al. (2021) [32]	Mixed Methods study.	Mozambique (rural)	Mental Health (Unhealthy Alcohol Use)	15 (qualitative) 45 (quantitative)	Psychiatric technicians and primary care practitioners	mHealth (smartphone application: mSBRIT*).	-mHealth was used in screening for unhealthy alcohol use by providing evidence-based questions and the calculation of risk. -mHealth was used in the treatment of unhealthy alcohol use through decision support.
Ojagbemi et al. (2022) [33]	Descriptive qualitative study	Nigeria (rural and urban)	Mental health	34	Nurses, community health officers and community health extension officers.	mHealth (smartphone application: Electronic version of the WHO* Mental Health Gap Action Programme intervention).	-mHealth was used in the treatment of common mental health disorders by guiding the management of mental health conditions.
Jacobs et al. (2020) [34]	Descriptive qualitative study	South Africa (not specified)	Mental health	34	Facility-based counsellors	mHealth (Telephone calls and WhatsApp Messenger).	-mHealth was used in the supervision of facility-based counsellors delivering psychosocial therapy for common mental health disorders through real-time responses using WhatsApp, and scheduled telephonic supervision.
Paddick et al. (2020) [35]	Cross-sectional study	Tanzania (rural)	Mental health	3011	CHWs	mHealth (smartphone application).	-mHealth was used in screening for dementia through administering a questionnaire.
Selohilwe et al. (2023) [36]	Qualitative study	South Africa (not specified)	Mental health (depression)	86	Lay health counsellors	mHealth (Telephone calls).	-mHealth was used in the supervision of lay health counsellors providing counselling for depression through the availability of supervisors telephonically when there was a need.
Triplett et al. (2023) [37]	Qualitative study	Kenya (not specified)	Mental health	27	Lay counsellors (teachers and community health volunteers)	mHealth (smart mobile phones used for communication through calling, WhatsApp Messenger, SMS* and images).	-mHealth was used in supervision through scheduling supervision appointments and advising on clinical matters.
Tsai et al. (2014) [38]	Cross-sectional analytical study	South Africa (peri-urban)	Mental Health	1144 (Study 1) 361 (Study 2)	CHWs	mHealth (smartphone application).	-mHealth was used in screening for antenatal depression through administering a questionnaire.
Vedanthan et al. (2019) [39]	Cluster randomised controlled trial	Kenya (rural)	Hypertension	1460	Community health volunteers	mHealth (smartphone linked to a decision support and electronic records system).	-Digital health was used for preventing hypertension through decision support for linkage to care.
Catley et al. (2022) [40]	Randomised controlled trial	South Africa (urban)	Type 2 diabetes and CVD risk factors.	494	CHWs	Video-based education and mHealth (SMS).	-Digital health was used for preventing Type 2 diabetes and CVD* risks through CHWs playing video sessions on physical activity and diet.

*BMI-Body Mass Index, CHW-Community health worker, CVD- Cardiovascular disease, mSBIRT -Screening, Brief Intervention, and Referral to Treatment, NCD- Non-communicable disease, NPHW- Non-physician Health Worker, SMS-Short message service, WHO-World Health Organization

### What are the characteristics of NPHWs using digital health for task-sharing in NCD prevention and management?

The NPHWs involved in using digital health varied across studies, with CHWs included in seven (46.7%) studies [26,29,31,35,38–40]. Lay health counsellors were included in another four (26.7%) studies [27,34,36,37]. Nurses were end users in two (13.3%) studies [28,33], while pharmacists [30], psychiatric technicians and primary care practitioners [32] also used digital health technologies in one study each.

Seven (46.7%) studies [26,29–32,35,38] indicated that NPHWs received training to use digital health technologies, while eight (53.3%) studies did not report information on training. The training duration ranged from two days [35] to nine days [27]. In two studies, training included experiential training [40], and apprenticeship [36].

### What type of digital health technologies were employed in task-sharing for preventing and managing NCDs?

mHealth was the commonly used digital health technology used in all included studies. In one study [40], video-based training was also used together with short message services (SMSs). In nine studies [26,29–33,35,38,39]—which described the use of mHealth—the NPHWs used smart mobile phone applications. The smart mobile phone applications entailed the use of software [31] on mobile phones that assisted with evidence-based information to guide the NPHWs [32]. Additionally, six studies [27,28,34,36,37,40], which described mHealth, noted the use of mobile phone features such as calling, imaging, and messaging.

### How are digital health technologies used in task-sharing for NCD prevention and management?

In six studies [26,27,29,30,33,39], digital health technologies were used for multiple functions. This review identified four key functions of digital health technologies in task-sharing for the prevention and management of NCDs. These functions include prevention, screening, treatment delivery, and supervision.

I. Digital health used in NCD prevention

Two studies [39,40] described the use of digital health by NPHWs to prevent NCDs. This included a weight reduction lifestyle intervention for Type 2 diabetes and cardiovascular disease (CVD) risk reduction delivered by CHWs who used video-based sessions [40]. The CHWs would set up and play video sessions on physical activity and diet with groups of community members [40]. Similarly, hypertension was prevented through linkage to care at primary health care facilities by CHWs equipped with smartphones, which enabled decision support and linkage to electronic health records [39]. In these primary healthcare facilities, nurses or clinical officers with whom CHWs shared tasks, provided hypertension management and confirmed the screening done by CHWs [39].

II. mHealth used in screening NCDs

Seven studies [26,28,29,31,32,35,38] reported the use of mHealth by NPHWs for screening NCDs. In Ghana, mHealth in the form of non-customized smartphone applications like WhatsApp and smartphone imaging was used for cervical cancer screening by nurses who collaborated with specialist gynecologists during quality assurance meetings held monthly [28]. Nurses would use digital health to take images when a mobile colposcopy was used in cervical cancer screenings and send images using WhatsApp to the specialist gynecologists for real-time decision support [28]. In six studies [26,29,31,32,35,38], NPHWs used customized smartphone applications to screen NCDs. In four studies [26,32,35,38], customized mHealth applications, including the Inuka App [26], the Screening Brief Intervention and Referral to Treatment (mSBRIT) App [32], were used to screen NCDs by providing evidence-based screening questions. For example, Tsai et al. [38] noted that the questions were based on the Edinburgh Postnatal Depression Scale, whereas Paddick et al. [35] used a validated questionnaire to assess cognitive and functional impairment. In addition, two studies [31,32] have shown that mobile applications (mSBRIT and Kobocollect App) can screen individuals by calculating a risk profile after the screening questions. In the case of the mSBRIT [32], the smart mobile phone application was used to support psychiatric technicians, CHWs, and primary health practitioners supervised and trained by specialists such as psychologists. Similarly, the smart mobile phone application -Kobocollect App- enabled the calculation of the Body Mass Index (BMI) as part of the risk assessment in the screening for CVD risks when tasks were shared between CHWs and NCD nurses working in community health centers [31]. In one study, the Muzima application screened for diabetes and hypertension through decision support [29].

III. mHealth used in delivering NCD treatment by NPHWs

In five studies [26,27,30,32,33], mHealth was used to deliver NCD treatment. Chibanda et al. [27] describe the use of mHealth to enable provider-to-patient communication, noting that “up to six SMSs and phone calls” were used to encourage problem-solving skills among patients receiving psychosocial support from lay health workers. The lay health workers were trained and supervised by health promotion officers and supported by psychiatrists [27]. Telephone calls were also used to follow patients who had missed their in-person psychosocial support sessions [27]. In three studies, smartphone applications were used to deliver mental health interventions by NPHWs [26,32,33]. Examples of these interventions comprised sessions of psychosocial therapy [26] and decision support in the form of discussion points based on outcomes from screening for unhealthy alcohol use [32]. The NPHWs found that using mHealth to deliver mental health treatment is feasible and acceptable [32]. In Nigeria, the smartphone application was based on the WHO Mental Health Gap Action Program, which also guided the management of various mental health conditions. NPHWs in Nigeria provided care under the supervision of senior nurses and general physicians [33]. Likewise, Nelissen et al. [30] described the use of a smartphone application by pharmacists to register patient records for patients with hypertension, enabling pharmacists to collaborate remotely with cardiologists.

IV. mHealth used in the supervision of NPHWs

Three studies [34,36,37] described the use of mHealth in the form of simple phone features, such as calling and WhatsApp Messenger, for supervision of NPHWs who provided mental health care. WhatsApp Messenger was used to supervise NPHWs by providing real-time responses beyond formal supervision [34,37], and could also be used to schedule supervision appointments [37]. In some cases, during counselling sessions, telephone calls were made to supervisors to seek advice [37]. Moreover, telephonic supervision was useful for hard-to-reach areas or when community unrest made it difficult to reach NPHWs [34]. In the three studies, mobile phones were used to augment in-person supervision [34,36,37].

**Fig 2:**
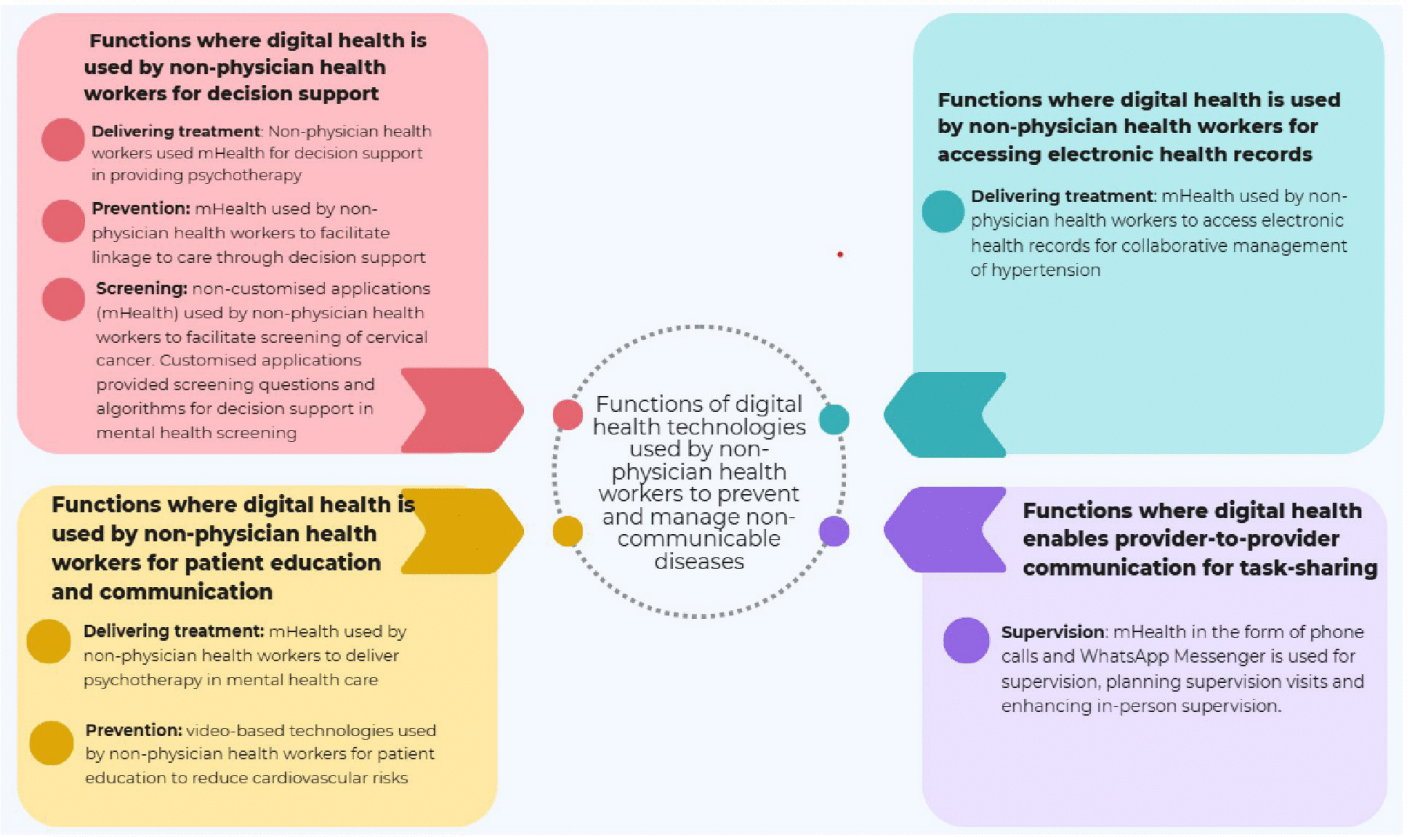
Technologies and their functions in task-sharing for non-communicable disease prevention and management.

In summary, mHealth was used by NPHWs for decision support in preventing, screening and delivering treatment. Digital health technologies were also used for patient education and communication to support prevention and treatment delivery. mHealth was also used to access patient electronic health records to support treatment delivery and to enable provider-to-provider communication for supervision of NPHWs. Fig 2. technologies and their functions in task-sharing for non-communicable disease prevention and management, summarizes the findings.

### How effective are digital health technologies used by NPHWs in improving the prevention and management of NCDs?

Four studies [26,27,39,40] reported patient outcomes when NPHWs use digital health technologies for task-sharing in preventing and managing NCDs. Two studies [26,27] described positive effects on patient outcomes when NPHWs used digital health technologies in the management of mental health conditions. Patients’ symptoms of common mental health problems improved over time when mHealth was used by community health volunteers [26]. Similarly, depressive symptoms and symptoms of common mental health conditions were also significantly lower in the intervention group than the control group when lay health counsellors administered a psychosocial intervention using mHealth [27]. The other two studies [39,40] reported neutral outcomes. Catley et al. [40] found that video-based training conducted by CHWs facilitated weight reduction for the prevention of Type 2 diabetes. However, there was no significant difference between the intervention and control groups in lowering glycated hemoglobin (HbA1c) levels, low-density lipoprotein (LDL) cholesterol levels, or blood pressure. In another study, Vedanthan et al. [39] reported neutral results, noting that while CHWs equipped with mHealth improved patient linkage to care for patients with hypertension, there was no difference in systolic blood pressure reduction between the control and intervention groups. The effectiveness of digital health technologies used by NPHWs in managing and preventing NCDs is summarized in Table 2.

**Table 2:** Effectiveness of digital health tools by NPHWs for the prevention and management of NCDs.

Study	Condition managed	Digital health technology used and (Intervention provided)	NPHW	Primary outcome measured	Primary outcome result
Chibanda et al. [27]	Mental health	mHealth through SMS*, to provide problem-solving skills as part of psychosocial therapy.	Lay health counsellors.	Primary outcome: the score on the SSQ-14* questionnaire, which measured symptoms of common mental health disorders.	The intervention group had fewer symptoms of common mental health disorders than the control group (3.81; 95%CI, 3.28 to 4.34 versus 8.90; 95% CI, 8.33 to 9.47) (p-value < 0.001). The intervention group had fewer symptoms of risk of depression than the control group (13.7% versus 49.9%) (p < 0.001).
Doukani et al. [26]	Mental health	mHealth was used to provide psychosocial therapy.	Community health volunteers.	Scores on the symptoms of CMD* (depression, anxiety) were measured using the WHO* SRQ-20*.	There was a significant (p < 0.001) decrease in symptoms of CMD among respondents over 3 months by 0.53 scores (95% CI: −3.954 to −2.636).
Catley et al. [40]	Type 2 Diabetes	Video-based training sessions to deliver lifestyle intervention for Type 2 Diabetes risk reduction.	CHWs*	The primary outcome was weight loss.	There was no significant difference (p = 0.71) in weight reduction between the intervention group, where weight change was −0.61% (95% CI: 1.22 to −0.01), and the control group, where weight change was −0.44% (95% CI = −1.06 to 0.18).
Vedanthan et al. [39]	Hypertension	mHealth in the form of a smartphone linked to decision support and electronic health records to improve linkage to care.	Community Health Volunteers.	The primary outcomes were linkage to care and change in systolic blood pressure.	There was a significant increase in linkage to care in both the smartphone arm (54%) and the usual care arm (50%), while the paper-based arm was 43%. There was a moderate difference in systolic blood pressure between the intervention group versus the usual care group (−13.1 mmHg versus −9.7).

*CHW-Community health workers, CMD-Common mental health disorders, NPHWs- Non-physician health workers, SMS-Short Message Service, SRQ-20-Self-reporting questionnaire-20, SSQ-14-Shona symptom questionnaire-14, WHO-World Health Organization.

### What affects optimal use of digital health technologies to support task-sharing for NCD prevention and management?

Studies reported challenges that affect the optimal use of digital health technologies by NPHWs. These included issues with low digital literacy skills among some NPHWs [28,29]. For example, nurses found it challenging to take pictures using smartphones during cervical cancer screening due to low digital literacy [28]. Additionally, participants found the forms on the mHealth application (Muzima) complicated for them to screen for diabetes and hypertension [29]. Challenges at the community level included poor internet connectivity and electricity outages for charging mobile devices [29,32,37].

## Discussion

This review included 15 studies that described the use of digital health technologies by NPHWs to prevent and manage NCDs in Africa. We found that mHealth (mobile health) was the predominant digital health technology across all studies, encompassing SMSs, mobile applications, electronic decision-support tools, and telehealth. Community health workers were the primary users (46.7%; n = 7) of digital health technologies. The digital health interventions implemented by NPHWs were used for prevention, screening, treatment delivery, and supervision, with tasks shared among various cadres in primary healthcare facilities and district hospitals. Additionally, digital health technologies used in NCD management were effective in providing treatment, while studies focusing on NCD prevention yielded neutral results. This review also highlights that digital health adoption was shaped by factors such as the adequacy of training provided to NPHWs, the availability of reliable internet connectivity, and access to electricity.

Digital health technologies are increasingly recognized as innovative tools for providing care [14,16]. Consistent with a previous scoping review, this study found that mHealth was the most used digital health technology among NPHWs, particularly CHWs, whose roles often involve door-to-door service delivery [41]. Our review also reported video-based tools used by NPHWs, but the limited range of technologies suggests that task-sharing in Africa has yet to integrate other innovations, such as AI, which the WHO recognizes as important for healthcare delivery [16].

Evidence from other settings shows that AI can be integrated into mobile applications to support health workers. For example, in France, AI-enabled mHealth chatbots were used as decision-support tools to expand cervical cancer screening through primary healthcare providers [42]. This study also described the use of customised mHealth applications, which aligns with evidence from other settings showing that tailored tools can enhance usability for CHWs with lower education levels [43]. Realizing these benefits in Africa requires context-sensitive strategies that address technological constraints and enabling conditions such as strong governance frameworks, investment in infrastructure, workforce training, and robust data systems.

This study identified four key functions of digital health technologies in NCD prevention and management, aligning with four of the twelve core functions described by Labrique et al. [44]: decision support through checklists and algorithms, provider-to-provider communication, patient education and communication, and access to electronic health records to support treatment. These functions enabled NPHWs to screen, educate, supervise, and treat patients more effectively, highlighting the potential of digital health to strengthen task-sharing for NCD care. However, functions such as human resource management, provider training, financial management, supply chain management, and data collection were beyond the scope of this study. Similar to previous findings, the use of sensors and point-of-care diagnostics integrated with mHealth remains largely unreported in low-resource settings in Africa [44]. Developing mHealth applications that incorporate these functions could enhance NPHWs’ capacity to prevent and manage NCDs more effectively.

Although this study identified only a limited range of digital health technologies and their functions, the findings are consistent with evidence from other LMICs. In India, NPHWs used customised mHealth applications to screen individuals for type 2 diabetes, hypertension, and mental health conditions by accessing electronic health records and providing structured screening questions [45]. Similarly, in Peru, mHealth was used by NPHWs to screen for mental health conditions via mobile questionnaires [46]. Systematic reviews from other LMICs also highlight the use of mHealth for health education, NCD prevention, and delivery of brief psychosocial interventions by CHWs [47,48]. When digital health is applied in task-sharing, there is evidence of improved patient outcomes; however, the certainty of evidence, particularly for mental health management by NPHWs, remains low [47]. Consistent with this, our study found neutral results for digital health in NCD prevention and positive outcomes in treatment, although we included only four studies to assess its effectiveness. Importantly, patient outcomes are influenced by factors beyond the health worker’s control, such as medication adherence and availability [49]. Future studies should therefore evaluate how digital health can enhance NPHWs’ effectiveness in NCD care.

The study also found that optimal use of digital health by NPHWs in NCD prevention and management is affected by low skill levels among NPHWs, inadequate electricity supply, and poor internet connectivity. The individual-level skill gaps align with those reported by Mishra et al. [48], who found low digital literacy among CHWs in LMICs. In our review, seven studies reported that NPHWs received training in digital technologies; however, low digital literacy may persist. Evidence suggests that poor digital literacy can be improved by involving end-users in the design of digital health technologies [43,50]. Inadequate electricity and poor internet connectivity are significant challenges in African healthcare systems, particularly in rural areas where infrastructure deficits are most pronounced [51].

Approximately 38% of African countries possess moderately mature digital health ecosystems, characterized by structured governance and ongoing investments [52]. However, many others remain at foundational or fragmented stages, limiting scalability and workforce readiness [52]. These infrastructural constraints hinder the effective deployment of digital health technologies for service delivery in the region [52]. African governments should strengthen infrastructure and ensure reliable service delivery, especially in underserved areas, to support scalable and effective digital health initiatives delivered by NPHWs.

Although we made efforts to ensure the robustness of our findings, this review has some limitations. Excluding grey literature and restricting the search to English-language publications may have reduced the breadth of the included studies. Nonetheless, the review provides valuable insights into current knowledge and highlights gaps in optimizing digital health for task-sharing in NCD prevention and management.

## Conclusion

As the NCD burden rises and health workforce challenges persist, it is critical to integrate and scale up digital technologies to enhance service delivery. Our review demonstrates that digital health, particularly mHealth, supports NPHWs in task-sharing for NCD prevention and management in Africa, providing functions for prevention, screening, treatment delivery, and supervision. The use of digital health was limited by low digital literacy, poor internet connectivity, and inadequate electricity supply. Effective implementation requires governance, infrastructure, workforce capacity, and robust data systems tailored to the contexts of African countries, alongside scaling up proven digital health solutions, which may have cost implications. This requires a whole-of-health system and whole-of-government approach, with regional and private partners aligned to national priorities to ensure sustainability.

## Supporting information

S1 PRISMA Checklist The PRISMA-ScR Checklist. (DOCX)

S1 Appendix The database search strategy. (PDF)

S2 Appendix The data abstraction tool. (PDF)
