# Supplementary material for: Use of digital health in task-sharing for prevention and management of non-communicable diseases in Africa: A scoping review
Source: PLOS Glob Public Health. 2026 Jul 24;6(7):e0006746. doi: 10.1371/journal.pgph.0006746 (PMC13399288; doi:10.1371/journal.pgph.0006746)
Supplement: S1 Appendix — (PDF) [file pgph.0006746.s002.pdf]

S1_Appendix

Use of Digital Health in Task-sharing for Prevention and Management of Non-Communicable Diseases in Africa: A Scoping Review

**The PubMed Search strategy**	
#5	(((#1) AND (#2)) AND (#3)) AND (#4),<br>2,892 Time 20:13:13
#1<br>Keyword: task sharing	"(((""task""[All Fields] AND (""share""[All Fields] OR ""shared""[All Fields] OR ""shares""[All Fields] OR ""sharing""[All Fields] OR ""sharings""[All Fields])) OR ((""distribute""[All Fields] OR ""distributed""[All Fields] OR ""distributer""[All Fields] OR ""distributers""[All Fields] OR ""distributes""[All Fields] OR ""distributing""[All Fields] OR ""distributional""[All Fields] OR ""distributions""[All Fields] OR ""supply and distribution""[MeSH Subheading] OR (""supply""[All Fields] AND ""distribution""[All Fields]) OR ""supply and distribution""[All Fields] OR ""distribution""[All Fields]) AND (""task s""[All Fields] OR ""tasking""[All Fields] OR ""taskings""[All Fields] OR ""tasks""[All Fields])) OR ((""allocate""[All Fields] OR ""allocated""[All Fields] OR ""allocates""[All Fields] OR ""allocating""[All Fields] OR ""allocation""[All Fields] OR ""allocational""[All Fields] OR ""allocations""[All Fields] OR ""allocative""[All Fields] OR ""allocator""[All Fields] OR ""allocators""[All Fields]) AND (""duties""[All Fields] OR ""dutiful""[All Fields] OR ""dutifulness""[All Fields])) OR (""task""[All Fields] AND (""distribute""[All Fields] OR ""distributed""[All Fields] OR ""distributer""[All Fields] OR ""distributers""[All Fields] OR ""distributes""[All Fields] OR ""distributing""[All Fields] OR ""distributional""[All Fields] OR ""distributions""[All Fields] OR ""supply and distribution""[MeSH Subheading] OR (""supply""[All Fields] AND ""distribution""[All Fields]) OR ""supply and distribution""[All Fields] OR ""distribution""[All Fields])) OR ((""share""[All Fields] OR ""shared""[All Fields] OR ""shares""[All Fields] OR ""sharing""[All Fields] OR ""sharings""[All Fields]) AND (""task s""[All Fields] OR ""tasking""[All Fields] OR ""taskings""[All Fields] OR ""tasks""[All Fields])) OR ((""allocate""[All Fields] OR ""allocated""[All Fields] OR ""allocates""[All Fields] OR ""allocating""[All Fields] OR ""allocation""[All Fields] OR ""allocational""[All Fields] OR ""allocations""[All Fields] OR ""allocative""[All Fields] OR ""allocator""[All Fields] OR ""allocators""[All Fields]) AND (""functional""[All Fields] OR ""functional s""[All Fields] OR ""functionalities""[All Fields] OR ""functionality""[All Fields] OR ""functionalization""[All Fields] OR ""functionalizations""[All Fields] OR ""functionalize""[All Fields] OR ""functionalized""[All Fields] OR ""functionalizes""[All Fields] OR ""functionalizing""[All Fields] OR ""functionally""[All Fields] OR ""functionals""[All Fields] OR ""functioned""[All Fields] OR ""functioning""[All Fields] OR ""functionings""[All Fields] OR ""functions""[All Fields] OR ""physiology""[MeSH Subheading] OR ""physiology""[All Fields] OR ""function""[All Fields] OR ""physiology""[MeSH Terms])) OR (""community health workers""[MeSH Terms] OR (""community""[All Fields] AND ""health""[All Fields] AND ""workers""[All Fields]) OR ""community health workers""[All Fields] OR (""community""[All Fields] AND ""health""[All Fields] AND ""worker""[All Fields]) OR ""community health worker""[All Fields]) OR (""cooperative behaviour""[All Fields] OR ""cooperative behavior""[MeSH Terms] OR (""cooperative""[All Fields] AND ""behavior""[All Fields]) OR ""cooperative behavior""[All Fields]) OR (""task shifting""[MeSH Terms] OR (""task""[All Fields] AND ""shifting""[All Fields]) OR ""task shifting""[All Fields]) OR (""non-specialist""[All Fields] AND (""health""[MeSH Terms] OR ""health""[All Fields] OR ""health s""[All Fields] OR ""healthful""[All Fields] OR ""healthfulness""[All Fields] OR ""healths""[All Fields]) AND (""occupational groups""[MeSH Terms] OR (""occupational""[All Fields] AND ""groups""[All Fields]) OR ""occupational groups""[All Fields] OR ""worker""[All Fields] OR ""workers""[All Fields] OR ""worker s""[All Fields])) OR ((""allocate""[All Fields] OR ""allocated""[All Fields] OR ""allocates""[All Fields] OR ""allocating""[All Fields] OR ""allocation""[All Fields] OR ""allocational""[All Fields] OR ""allocations""[All Fields] OR ""allocative""[All Fields] OR ""allocator""[All Fields] OR ""allocators""[All Fields]) AND (""task s""[All Fields] OR ""tasking""[All Fields] OR ""taskings""[All Fields] OR ""tasks""[All Fields])) OR (""non-physician""[All Fields] AND (""health""[MeSH Terms] OR ""health""[All Fields] OR

	""health s""[All Fields] OR ""healthful""[All Fields] OR ""healthfulness""[All Fields] OR ""healths""[All Fields]) AND (""occupational groups""[MeSH Terms] OR (""occupational""[All Fields] AND ""groups""[All Fields]) OR ""occupational groups""[All Fields] OR ""worker""[All Fields] OR ""workers""[All Fields] OR ""worker s""[All Fields])) OR ((""distribute""[All Fields] OR ""distributed""[All Fields] OR ""distributer""[All Fields] OR ""distributers""[All Fields] OR ""distributes""[All Fields] OR ""distributing""[All Fields] OR ""distributional""[All Fields] OR ""distributions""[All Fields] OR ""supply and distribution""[MeSH Subheading] OR (""supply""[All Fields] AND ""distribution""[All Fields]) OR ""supply and distribution""[All Fields] OR ""distribution""[All Fields]) AND (""duties""[All Fields] OR ""dutiful""[All Fields] OR ""dutifulness""[All Fields])) OR (""task""[All Fields] AND (""share""[All Fields] OR ""shared""[All Fields] OR ""shares""[All Fields] OR ""sharing""[All Fields] OR ""sharings""[All Fields])) OR ((""share""[All Fields] OR ""shared""[All Fields] OR ""shares""[All Fields] OR ""sharing""[All Fields] OR ""sharings""[All Fields]) AND (""task s""[All Fields] OR ""tasking""[All Fields] OR ""taskings""[All Fields] OR ""tasks""[All Fields])) OR ((""shift""[All Fields] OR ""shifted""[All Fields] OR ""shifting""[All Fields] OR ""shiftings""[All Fields] OR ""shifts""[All Fields]) AND (""task s""[All Fields] OR ""tasking""[All Fields] OR ""taskings""[All Fields] OR ""tasks""[All Fields])) OR (""responsabilities""[All Fields] OR ""responsability""[All Fields] OR ""responsibilities""[All Fields] OR ""responsibility""[All Fields] OR ""responsible""[All Fields] OR ""responsibles""[All Fields]) OR (""lay""[All Fields] AND (""health""[MeSH Terms] OR ""health""[All Fields] OR ""health s""[All Fields] OR ""healthful""[All Fields] OR ""healthfulness""[All Fields] OR ""healths""[All Fields]) AND (""occupational groups""[MeSH Terms] OR (""occupational""[All Fields] AND ""groups""[All Fields]) OR ""occupational groups""[All Fields] OR ""worker""[All Fields] OR ""workers""[All Fields] OR ""worker s""[All Fields])) OR (""lay""[All Fields] AND (""counsellor""[All Fields] OR ""counselors""[MeSH Terms] OR ""counselors""[All Fields] OR ""counselor""[All Fields] OR ""counseling""[MeSH Terms] OR ""counseling""[All Fields] OR ""counsellor s""[All Fields] OR ""counsellors""[All Fields] OR ""counselor s""[All Fields])) OR (""community health workers""[MeSH Terms] OR (""community""[All Fields] AND ""health""[All Fields] AND ""workers""[All Fields]) OR ""community health workers""[All Fields] OR (""village""[All Fields] AND ""health""[All Fields] AND ""worker""[All Fields]) OR ""village health worker""[All Fields]) OR ((""internet""[MeSH Terms] OR ""internet""[All Fields] OR ""internet s""[All Fields] OR ""internets""[All Fields]) AND (""medic""[All Fields] OR ""medical""[All Fields] OR ""medicalization""[MeSH Terms] OR ""medicalization""[All Fields] OR ""medicalizations""[All Fields] OR ""medicalize""[All Fields] OR ""medicalized""[All Fields] OR ""medicalizes""[All Fields] OR ""medicalizing""[All Fields] OR ""medically""[All Fields] OR ""medicals""[All Fields] OR ""medicated""[All Fields] OR ""medication s""[All Fields] OR ""medics""[All Fields] OR ""pharmaceutical preparations""[Supplementary Concept] OR ""pharmaceutical preparations""[All Fields] OR ""medication""[All Fields] OR ""pharmaceutical preparations""[MeSH Terms] OR (""pharmaceutical""[All Fields] AND ""preparations""[All Fields]) OR ""medications""[All Fields]) AND (""thing""[All Fields] OR ""things""[All Fields]))) 991,436 Time 19:35:11
#2<br>Digital health	(""digital health""[MeSH Terms] OR (""digital""[All Fields] AND ""health""[All Fields]) OR ""digital health""[All Fields] OR (""telemedicine""[MeSH Terms] OR ""telemedicine""[All Fields] OR ""ehealth""[All Fields]) OR ""e-health""[All Fields] OR ((""electronical""[All Fields] OR ""electronically""[All Fields] OR ""electronics""[MeSH Terms] OR ""electronics""[All Fields] OR ""electronic""[All Fields]) AND (""health""[MeSH Terms] OR ""health""[All Fields] OR ""health s""[All Fields] OR ""healthful""[All Fields] OR ""healthfulness""[All Fields] OR ""healths""[All Fields])) OR (""digital technology""[MeSH Terms] OR (""digital""[All Fields] AND ""technology""[All Fields]) OR ""digital technology""[All Fields] OR (""digital""[All Fields] AND ""technologies""[All Fields]) OR ""digital technologies""[All Fields]) OR ((""digitalisation""[All Fields] OR ""digitalised""[All Fields] OR ""digitalization""[All Fields] OR ""digitalize""[All Fields] OR ""digitalized""[All Fields] OR ""digitalizer""[All Fields] OR ""digitalizing""[All Fields] OR ""digitally""[All Fields] OR ""digitals""[All Fields] OR ""digitization""[All Fields] OR ""digitizations""[All Fields] OR ""digitize""[All Fields] OR ""digitized""[All Fields] OR ""digitizer""[All Fields] OR ""digitizers""[All Fields] OR ""digitizes""[All Fields] OR ""digitizing""[All Fields] OR ""radiographic image enhancement""[MeSH Terms] OR (""radiographic""[All Fields] AND ""image""[All Fields] AND ""enhancement""[All Fields]) OR ""radiographic image enhancement""[All Fields] OR ""digital""[All Fields]) AND (""intervention

	s""[All Fields] OR ""interventions""[All Fields] OR ""interventive""[All Fields] OR ""methods""[MeSH Terms] OR ""methods""[All Fields] OR ""intervention""[All Fields] OR ""interventional""[All Fields])) OR ((""electronical""[All Fields] OR ""electronically""[All Fields] OR ""electronics""[MeSH Terms] OR ""electronics""[All Fields] OR ""electronic""[All Fields]) AND ""care""[All Fields]) OR (""telemedicine""[MeSH Terms] OR ""telemedicine""[All Fields] OR ""telemedicine s""[All Fields]) OR (""tele""[All Fields] AND (""medicin""[All Fields] OR ""medicinal""[All Fields] OR ""medicinally""[All Fields] OR ""medicinals""[All Fields] OR ""medicine""[MeSH Terms] OR ""medicine""[All Fields] OR ""medicine s""[All Fields] OR ""medicines""[All Fields])) OR (""telehealth s""[All Fields] OR ""telemedicine""[MeSH Terms] OR ""telemedicine""[All Fields] OR ""telehealth""[All Fields]) OR (""tele""[All Fields] AND (""health""[MeSH Terms] OR ""health""[All Fields] OR ""health s""[All Fields] OR ""healthful""[All Fields] OR ""healthfulness""[All Fields] OR ""healths""[All Fields])) OR (""telemedicine""[MeSH Terms] OR ""telemedicine""[All Fields] OR (""tele""[All Fields] AND ""care""[All Fields]) OR ""tele care""[All Fields]) OR (""tele""[All Fields] AND (""monitor""[All Fields] OR ""monitor s""[All Fields] OR ""monitorable""[All Fields] OR ""monitored""[All Fields] OR ""monitoring""[All Fields] OR ""monitoring s""[All Fields] OR ""monitorings""[All Fields] OR ""monitorization""[All Fields] OR ""monitorize""[All Fields] OR ""monitorized""[All Fields] OR ""monitors""[All Fields])) OR (""tele""[All Fields] AND (""consultancies""[All Fields] OR ""consultancy""[All Fields] OR ""consultant s""[All Fields] OR ""consultants""[MeSH Terms] OR ""consultants""[All Fields] OR ""consultant""[All Fields] OR ""consultative""[All Fields] OR ""consulter""[All Fields] OR ""consulters""[All Fields] OR ""referral and consultation""[MeSH Terms] OR (""referral""[All Fields] AND ""consultation""[All Fields]) OR ""referral and consultation""[All Fields] OR ""consult""[All Fields] OR ""consultation""[All Fields] OR ""consultations""[All Fields] OR ""consulted""[All Fields] OR ""consulting""[All Fields] OR ""consults""[All Fields])) OR (""remote consultation""[MeSH Terms] OR (""remote""[All Fields] AND ""consultation""[All Fields]) OR ""remote consultation""[All Fields] OR ""teleconsultation""[All Fields] OR ""teleconsultations""[All Fields] OR ""teleconsult""[All Fields] OR ""teleconsultant""[All Fields] OR ""teleconsultants""[All Fields] OR ""teleconsultative""[All Fields] OR ""teleconsulting""[All Fields] OR ""teleconsults""[All Fields]) OR ((""video s""[All Fields] OR ""videoed""[All Fields] OR ""videotape recording""[MeSH Terms] OR (""videotape""[All Fields] AND ""recording""[All Fields]) OR ""videotape recording""[All Fields] OR ""video""[All Fields] OR ""videos""[All Fields]) AND (""consultancies""[All Fields] OR ""consultancy""[All Fields] OR ""consultant s""[All Fields] OR ""consultants""[MeSH Terms] OR ""consultants""[All Fields] OR ""consultant""[All Fields] OR ""consultative""[All Fields] OR ""consulter""[All Fields] OR ""consulters""[All Fields] OR ""referral and consultation""[MeSH Terms] OR (""referral""[All Fields] AND ""consultation""[All Fields]) OR ""referral and consultation""[All Fields] OR ""consult""[All Fields] OR ""consultation""[All Fields] OR ""consultations""[All Fields] OR ""consulted""[All Fields] OR ""consulting""[All Fields] OR ""consults""[All Fields])) OR (""text messaging""[MeSH Terms] OR (""text""[All Fields] AND ""messaging""[All Fields]) OR ""text messaging""[All Fields] OR (""text""[All Fields] AND ""message""[All Fields]) OR ""text message""[All Fields]) OR (""text messaging""[MeSH Terms] OR (""text""[All Fields] AND ""messaging""[All Fields]) OR ""text messaging""[All Fields] OR ""text""[All Fields]) OR (""telemedicine""[MeSH Terms] OR ""telemedicine""[All Fields] OR (""mobile""[All Fields] AND ""health""[All Fields]) OR ""mobile health""[All Fields]) OR (""mhealth s""[All Fields] OR ""telemedicine""[MeSH Terms] OR ""telemedicine""[All Fields] OR ""mhealth""[All Fields]) OR (""m""[All Fields] AND (""health""[MeSH Terms] OR ""health""[All Fields] OR ""health s""[All Fields] OR ""healthful""[All Fields] OR ""healthfulness""[All Fields] OR ""healths""[All Fields])) OR ""m-health""[All Fields] OR (""android s""[All Fields] OR ""androids""[All Fields] OR ""methyltestosterone""[Supplementary Concept] OR ""methyltestosterone""[All Fields] OR ""android""[All Fields] OR ""methyltestosterone""[MeSH Terms]) OR (""australas plant pathol""[Journal] OR ""app""[All Fields]) OR (""audio""[All Fields] OR ""audios""[All Fields]) OR (""cell phone""[MeSH Terms] OR (""cell""[All Fields] AND ""phone""[All Fields]) OR ""cell phone""[All Fields] OR ""cellphone""[All Fields] OR ""cellphones""[All Fields]) OR (""cell phone""[MeSH Terms] OR (""cell""[All Fields] AND ""phone""[All Fields]) OR ""cell phone""[All Fields]) OR (""computability""[All Fields] OR ""computable""[All Fields] OR ""computating""[All Fields] OR ""computation""[All Fields] OR ""computational""[All Fields] OR ""computations""[All Fields] OR ""compute""[All Fields] OR

	""computed""[All Fields] OR ""computer s""[All Fields] OR ""computers""[MeSH Terms] OR ""computers""[All Fields] OR ""computer""[All Fields] OR ""computes""[All Fields] OR ""computing""[All Fields] OR ""computional""[All Fields]) OR (""mobile""[All Fields] OR ""mobiles""[All Fields]) OR ""multi-media""[All Fields] OR (""computers, handheld""[MeSH Terms] OR (""computers""[All Fields] AND ""handheld""[All Fields]) OR ""handheld computers""[All Fields] OR (""personal""[All Fields] AND ""digital""[All Fields] AND ""assistant""[All Fields]) OR ""personal digital assistant""[All Fields]) OR (""smart mater struct""[Journal] OR ""sms""[All Fields]) OR (""social media""[MeSH Terms] OR (""social""[All Fields] AND ""media""[All Fields]) OR ""social media""[All Fields]) OR (""software""[MeSH Terms] OR ""software""[All Fields] OR ""software s""[All Fields] OR ""softwares""[All Fields]) OR ""telecomm""[All Fields] OR ""etherapy""[All Fields] OR (""information technology""[MeSH Terms] OR (""information""[All Fields] AND ""technology""[All Fields]) OR ""information technology""[All Fields]) OR ((""instant""[All Fields] OR ""instants""[All Fields]) AND (""message""[All Fields] OR ""message s""[All Fields] OR ""messaged""[All Fields] OR ""messages""[All Fields] OR ""messaging""[All Fields])) OR (""internet""[MeSH Terms] OR ""internet""[All Fields] OR ""internet s""[All Fields] OR ""internets""[All Fields]) OR ""i-pad""[All Fields] OR ""ipad""[All Fields] OR (""iphone""[All Fields] OR ""iphone s""[All Fields] OR ""iphones""[All Fields]) OR ""i-phone""[All Fields] OR (""mp3 player""[MeSH Terms] OR ""mp3 player""[All Fields] OR ""i pod""[All Fields]) OR ""web""[All Fields] OR (""smartphone""[MeSH Terms] OR ""smartphone""[All Fields] OR (""smart""[All Fields] AND ""phone""[All Fields]) OR ""smart phone""[All Fields]) OR (""cell phone""[MeSH Terms] OR (""cell""[All Fields] AND ""phone""[All Fields]) OR ""cell phone""[All Fields] OR (""mobile""[All Fields] AND ""phone""[All Fields]) OR ""mobile phone""[All Fields]) OR (""electronic health records""[MeSH Terms] OR (""electronic""[All Fields] AND ""health""[All Fields] AND ""records""[All Fields]) OR ""electronic health records""[All Fields] OR (""electronic""[All Fields] AND ""health""[All Fields] AND ""record""[All Fields]) OR ""electronic health record""[All Fields]) OR (""health records, personal""[MeSH Terms] OR (""health""[All Fields] AND ""records""[All Fields] AND ""personal""[All Fields]) OR ""personal health records""[All Fields] OR (""personal""[All Fields] AND ""health""[All Fields] AND ""record""[All Fields]) OR ""personal health record""[All Fields]) OR (""electronic health records""[MeSH Terms] OR (""electronic""[All Fields] AND ""health""[All Fields] AND ""records""[All Fields]) OR ""electronic health records""[All Fields] OR (""electronic""[All Fields] AND ""medical""[All Fields] AND ""record""[All Fields]) OR ""electronic medical record""[All Fields]) OR (""electronic health records""[MeSH Terms] OR (""electronic""[All Fields] AND ""health""[All Fields] AND ""records""[All Fields]) OR ""electronic health records""[All Fields] OR (""computer""[All Fields] AND ""based""[All Fields] AND ""patient""[All Fields] AND ""record""[All Fields]) OR ""computer based patient record""[All Fields]) OR (""health information systems""[MeSH Terms] OR (""health""[All Fields] AND ""information""[All Fields] AND ""systems""[All Fields]) OR ""health information systems""[All Fields] OR (""health""[All Fields] AND ""information""[All Fields] AND ""system""[All Fields]) OR ""health information system""[All Fields]) OR ((""internet""[MeSH Terms] OR ""internet""[All Fields] OR ""internet s""[All Fields] OR ""internets""[All Fields]) AND (""medic""[All Fields] OR ""medical""[All Fields] OR ""medicalization""[MeSH Terms] OR ""medicalization""[All Fields] OR ""medicalizations""[All Fields] OR ""medicalize""[All Fields] OR ""medicalized""[All Fields] OR ""medicalizes""[All Fields] OR ""medicalizing""[All Fields] OR ""medically""[All Fields] OR ""medicals""[All Fields] OR ""medicated""[All Fields] OR ""medication s""[All Fields] OR ""medics""[All Fields] OR ""pharmaceutical preparations""[Supplementary Concept] OR ""pharmaceutical preparations""[All Fields] OR ""medication""[All Fields] OR ""pharmaceutical preparations""[MeSH Terms] OR (""pharmaceutical""[All Fields] AND ""preparations""[All Fields]) OR ""medications""[All Fields]) AND (""thing""[All Fields] OR ""things""[All Fields])) OR (""robot""[All Fields] OR ""robot s""[All Fields] OR ""robotically""[All Fields] OR ""robotics""[MeSH Terms] OR ""robotics""[All Fields] OR ""robotic""[All Fields] OR ""robotization""[All Fields] OR ""robotized""[All Fields] OR ""robots""[All Fields]) OR (""artificial intelligence""[MeSH Terms] OR (""artificial""[All Fields] AND ""intelligence""[All Fields]) OR ""artificial intelligence""[All Fields]) OR ""e-pharmacy""[All Fields]) 5,049,389 Time 19:45:15
#3	(""noncommunicable diseases""[MeSH Terms] OR (""noncommunicable""[All Fields] AND ""diseases""[All Fields]) OR ""noncommunicable diseases""[All Fields] OR (""non""[All Fields] AND ""communicable""[All Fields] AND ""diseases""[All Fields]) OR ""non communicable

Keyword: non-communicable disease	diseases""[All Fields] OR (""chronic disease""[MeSH Terms] OR (""chronic""[All Fields] AND ""disease""[All Fields]) OR ""chronic disease""[All Fields] OR (""chronic""[All Fields] AND ""conditions""[All Fields]) OR ""chronic conditions""[All Fields]) OR ((""chronic""[All Fields] OR ""chronical""[All Fields] OR ""chronically""[All Fields] OR ""chronicities""[All Fields] OR ""chronicity""[All Fields] OR ""chronicization""[All Fields] OR ""chronics""[All Fields]) AND (""health""[MeSH Terms] OR ""health""[All Fields] OR ""health s""[All Fields] OR ""healthful""[All Fields] OR ""healthfulness""[All Fields] OR ""healths""[All Fields]) AND (""problem""[All Fields] OR ""problem s""[All Fields] OR ""problems""[All Fields])) OR (""chronic disease""[MeSH Terms] OR (""chronic""[All Fields] AND ""disease""[All Fields]) OR ""chronic disease""[All Fields] OR (""chronic""[All Fields] AND ""diseases""[All Fields]) OR ""chronic diseases""[All Fields]) OR (""hypertense""[All Fields] OR ""hypertension""[MeSH Terms] OR ""hypertension""[All Fields] OR ""hypertension s""[All Fields] OR ""hypertensions""[All Fields] OR ""hypertensive""[All Fields] OR ""hypertensive s""[All Fields] OR ""hypertensives""[All Fields]) OR (""arteriosclerosis""[MeSH Terms] OR ""arteriosclerosis""[All Fields] OR ""arterioscleroses""[All Fields]) OR (""cardiovascular diseases""[MeSH Terms] OR (""cardiovascular""[All Fields] AND ""diseases""[All Fields]) OR ""cardiovascular diseases""[All Fields] OR (""cardiovascular""[All Fields] AND ""disease""[All Fields]) OR ""cardiovascular disease""[All Fields]) OR (""blood pressure""[MeSH Terms] OR (""blood""[All Fields] AND ""pressure""[All Fields]) OR ""blood pressure""[All Fields] OR ""blood pressure determination""[MeSH Terms] OR (""blood""[All Fields] AND ""pressure""[All Fields] AND ""determination""[All Fields]) OR ""blood pressure determination""[All Fields] OR ""arterial pressure""[MeSH Terms] OR (""arterial""[All Fields] AND ""pressure""[All Fields]) OR ""arterial pressure""[All Fields]) OR (""diabete""[All Fields] OR ""diabetes mellitus""[MeSH Terms] OR (""diabetes""[All Fields] AND ""mellitus""[All Fields]) OR ""diabetes mellitus""[All Fields] OR ""diabetes""[All Fields] OR ""diabetes insipidus""[MeSH Terms] OR (""diabetes""[All Fields] AND ""insipidus""[All Fields]) OR ""diabetes insipidus""[All Fields] OR ""diabetic""[All Fields] OR ""diabetics""[All Fields] OR ""diabets""[All Fields]) OR (""diabetes insipidus""[MeSH Terms] OR (""diabetes""[All Fields] AND ""insipidus""[All Fields]) OR ""diabetes insipidus""[All Fields]) OR (""diabetes, gestational""[MeSH Terms] OR (""diabetes""[All Fields] AND ""gestational""[All Fields]) OR ""gestational diabetes""[All Fields] OR (""gestational""[All Fields] AND ""diabetes""[All Fields])) OR (""hyperglycaemia""[All Fields] OR ""hyperglycemia""[MeSH Terms] OR ""hyperglycemia""[All Fields] OR ""hyperglycaemias""[All Fields] OR ""hyperglycemias""[All Fields] OR ""hyperglycemia s""[All Fields]) OR (""blood glucose""[Supplementary Concept] OR ""blood glucose""[All Fields] OR ""blood glucose""[MeSH Terms] OR (""blood""[All Fields] AND ""glucose""[All Fields])) OR (""diabetes mellitus, type 2""[MeSH Terms] OR ""type 2 diabetes mellitus""[All Fields] OR ""diabetes mellitus type 2""[All Fields]) OR (""diabetes mellitus, type 2""[MeSH Terms] OR ""type 2 diabetes mellitus""[All Fields] OR ""type 2 diabetes""[All Fields]) OR (""diabetes mellitus, type 1""[MeSH Terms] OR ""type 1 diabetes mellitus""[All Fields] OR ""type 1 diabetes""[All Fields]) OR (""diabetes mellitus, type 1""[MeSH Terms] OR ""type 1 diabetes mellitus""[All Fields] OR ""diabetes mellitus type 1""[All Fields]) OR (""neoplasm s""[All Fields] OR ""neoplasms""[MeSH Terms] OR ""neoplasms""[All Fields] OR ""neoplasm""[All Fields]) OR (""cancer s""[All Fields] OR ""cancerated""[All Fields] OR ""canceration""[All Fields] OR ""cancerization""[All Fields] OR ""cancerized""[All Fields] OR ""cancerous""[All Fields] OR ""neoplasms""[MeSH Terms] OR ""neoplasms""[All Fields] OR ""cancer""[All Fields] OR ""cancers""[All Fields]) OR (""uterine cervical neoplasms""[MeSH Terms] OR (""uterine""[All Fields] AND ""cervical""[All Fields] AND ""neoplasms""[All Fields]) OR ""uterine cervical neoplasms""[All Fields] OR (""cervical""[All Fields] AND ""cancer""[All Fields]) OR ""cervical cancer""[All Fields]) OR (""uterine cervical neoplasms""[MeSH Terms] OR (""uterine""[All Fields] AND ""cervical""[All Fields] AND ""neoplasms""[All Fields]) OR ""uterine cervical neoplasms""[All Fields] OR (""cancer""[All Fields] AND ""cervix""[All Fields]) OR ""cancer of the cervix""[All Fields]) OR (""breast neoplasms""[MeSH Terms] OR (""breast""[All Fields] AND ""neoplasms""[All Fields]) OR ""breast neoplasms""[All Fields] OR (""breast""[All Fields] AND ""cancer""[All Fields]) OR ""breast cancer""[All Fields]) OR (""malign""[All Fields] OR ""malignance""[All Fields] OR ""malignances""[All Fields] OR ""malignant""[All Fields] OR ""malignants""[All Fields] OR ""malignities""[All Fields] OR ""malignity""[All Fields] OR ""malignization""[All Fields] OR ""malignized""[All Fields] OR ""maligns""[All Fields] OR ""neoplasms""[MeSH Terms] OR ""neoplasms""[All Fields] OR ""malignancies""[All Fields] OR

	""malignancy""[All Fields]) OR (""cysts""[MeSH Terms] OR ""cysts""[All Fields] OR ""cyst""[All Fields] OR ""neurofibroma""[MeSH Terms] OR ""neurofibroma""[All Fields] OR ""neurofibromas""[All Fields] OR ""tumor s""[All Fields] OR ""tumoral""[All Fields] OR ""tumorous""[All Fields] OR ""tumour""[All Fields] OR ""neoplasms""[MeSH Terms] OR ""neoplasms""[All Fields] OR ""tumor""[All Fields] OR ""tumour s""[All Fields] OR ""tumoural""[All Fields] OR ""tumourous""[All Fields] OR ""tumours""[All Fields] OR ""tumors""[All Fields]) OR (""prostatic neoplasms""[MeSH Terms] OR (""prostatic""[All Fields] AND ""neoplasms""[All Fields]) OR ""prostatic neoplasms""[All Fields] OR (""prostate""[All Fields] AND ""cancer""[All Fields]) OR ""prostate cancer""[All Fields]) OR (""asthma""[MeSH Terms] OR ""asthma""[All Fields] OR ""asthmas""[All Fields] OR ""asthma s""[All Fields]) OR (""respiratory tract diseases""[MeSH Terms] OR (""respiratory""[All Fields] AND ""tract""[All Fields] AND ""diseases""[All Fields]) OR ""respiratory tract diseases""[All Fields] OR (""respiratory""[All Fields] AND ""disorder""[All Fields]) OR ""respiratory disorder""[All Fields] OR ""respiration disorders""[MeSH Terms] OR (""respiration""[All Fields] AND ""disorders""[All Fields]) OR ""respiration disorders""[All Fields]) OR (""asthma""[MeSH Terms] OR ""asthma""[All Fields] OR (""bronchial""[All Fields] AND ""asthma""[All Fields]) OR ""bronchial asthma""[All Fields]) OR ((""lung diseases""[MeSH Terms] OR (""lung""[All Fields] AND ""diseases""[All Fields]) OR ""lung diseases""[All Fields] OR (""pulmonary""[All Fields] AND ""disease""[All Fields]) OR ""pulmonary disease""[All Fields]) AND (""chronic""[All Fields] OR ""chronical""[All Fields] OR ""chronically""[All Fields] OR ""chronicities""[All Fields] OR ""chronicity""[All Fields] OR ""chronicization""[All Fields] OR ""chronics""[All Fields]) AND (""obstruct""[All Fields] OR ""obstructed""[All Fields] OR ""obstructing""[All Fields] OR ""obstruction""[All Fields] OR ""obstructions""[All Fields] OR ""obstructive""[All Fields] OR ""obstructs""[All Fields])) OR ((""chronic""[All Fields] OR ""chronical""[All Fields] OR ""chronically""[All Fields] OR ""chronicities""[All Fields] OR ""chronicity""[All Fields] OR ""chronicization""[All Fields] OR ""chronics""[All Fields]) AND (""obstruct""[All Fields] OR ""obstructed""[All Fields] OR ""obstructing""[All Fields] OR ""obstruction""[All Fields] OR ""obstructions""[All Fields] OR ""obstructive""[All Fields] OR ""obstructs""[All Fields]) AND (""lung diseases""[MeSH Terms] OR (""lung""[All Fields] AND ""diseases""[All Fields]) OR ""lung diseases""[All Fields] OR (""pulmonary""[All Fields] AND ""disorder""[All Fields]) OR ""pulmonary disorder""[All Fields])) OR (""mental health""[MeSH Terms] OR (""mental""[All Fields] AND ""health""[All Fields]) OR ""mental health""[All Fields]) OR (""psychosocial support systems""[MeSH Terms] OR (""psychosocial""[All Fields] AND ""support""[All Fields] AND ""systems""[All Fields]) OR ""psychosocial support systems""[All Fields] OR (""psychosocial""[All Fields] AND ""support""[All Fields]) OR ""psychosocial support""[All Fields]) OR (""psychosocial intervention""[MeSH Terms] OR (""psychosocial""[All Fields] AND ""intervention""[All Fields]) OR ""psychosocial intervention""[All Fields]) OR (""psychotherapie""[All Fields] OR ""psychotherapy""[MeSH Terms] OR ""psychotherapy""[All Fields] OR ""psychotherapies""[All Fields] OR ""psychotherapy s""[All Fields]) OR (""epilepsie""[All Fields] OR ""epilepsy""[MeSH Terms] OR ""epilepsy""[All Fields] OR ""epilepsies""[All Fields] OR ""epilepsy s""[All Fields]) OR (""depressed""[All Fields] OR ""depression""[MeSH Terms] OR ""depression""[All Fields] OR ""depressions""[All Fields] OR ""depression s""[All Fields] OR ""depressive disorder""[MeSH Terms] OR (""depressive""[All Fields] AND ""disorder""[All Fields]) OR ""depressive disorder""[All Fields] OR ""depressivity""[All Fields] OR ""depressive""[All Fields] OR ""depressively""[All Fields] OR ""depressiveness""[All Fields] OR ""depressives""[All Fields]) OR (""depression""[MeSH Terms] OR ""depression""[All Fields] OR (""depressive""[All Fields] AND ""symptoms""[All Fields]) OR ""depressive symptoms""[All Fields]) OR (""schizophrenia""[MeSH Terms] OR ""schizophrenia""[All Fields] OR ""schizophrenias""[All Fields] OR ""schizophrenia s""[All Fields]) OR (""mental disorders""[MeSH Terms] OR (""mental""[All Fields] AND ""disorders""[All Fields]) OR ""mental disorders""[All Fields])) 14,124,530 Time 19:53
#4<br>Keyword<br>Africa	(""algeria""[MeSH Terms] OR ""algeria""[All Fields] OR (""angola""[MeSH Terms] OR ""angola""[All Fields] OR ""angola s""[All Fields]) OR (""benin""[MeSH Terms] OR ""benin""[All Fields] OR ""benin s""[All Fields]) OR (""botswana""[MeSH Terms] OR ""botswana""[All Fields] OR ""botswana s""[All Fields]) OR (""burkina faso""[MeSH Terms] OR (""burkina""[All Fields] AND ""faso""[All Fields]) OR ""burkina faso""[All Fields]) OR (""burundi""[MeSH Terms] OR ""burundi""[All Fields]) OR (""carbo""[All Fields] AND (""verde""[All Fields] OR ""verdes""[All Fields])) OR (""cameroon""[MeSH Terms] OR ""cameroon""[All Fields] OR ""cameroons""[All

	Fields] OR ""cameroon s""[All Fields]) OR (""central african republic""[MeSH Terms] OR (""central""[All Fields] AND ""african""[All Fields] AND ""republic""[All Fields]) OR ""central african republic""[All Fields]) OR (""chad""[MeSH Terms] OR ""chad""[All Fields]) OR (""comoros""[MeSH Terms] OR ""comoros""[All Fields] OR ""comoro""[All Fields]) OR ((""democrat""[All Fields] OR ""democratic""[All Fields] OR ""democratically""[All Fields] OR ""democratization""[All Fields] OR ""democratize""[All Fields] OR ""democratized""[All Fields] OR ""democratizing""[All Fields] OR ""democrats""[All Fields]) AND (""republic""[All Fields] OR ""republic s""[All Fields] OR ""republics""[All Fields]) AND (""congo""[MeSH Terms] OR ""congo""[All Fields])) OR (""cote d ivoire""[MeSH Terms] OR (""cote""[All Fields] AND ""d ivoire""[All Fields]) OR ""cote d ivoire""[All Fields]) OR (""djibouti""[MeSH Terms] OR ""djibouti""[All Fields]) OR (""egypt""[MeSH Terms] OR ""egypt""[All Fields] OR ""egypt s""[All Fields]) OR (""equatorial guinea""[MeSH Terms] OR (""equatorial""[All Fields] AND ""guinea""[All Fields]) OR ""equatorial guinea""[All Fields]) OR (""eritrea""[MeSH Terms] OR ""eritrea""[All Fields]) OR (""eswatini""[MeSH Terms] OR ""eswatini""[All Fields]) OR (""ethiopia""[MeSH Terms] OR ""ethiopia""[All Fields] OR ""ethiopia s""[All Fields]) OR (""gabon""[MeSH Terms] OR ""gabon""[All Fields]) OR (""gambia""[MeSH Terms] OR ""gambia""[All Fields] OR ""gambia s""[All Fields]) OR (""south sudan""[MeSH Terms] OR (""south""[All Fields] AND ""sudan""[All Fields]) OR ""south sudan""[All Fields]) OR (""sudan""[MeSH Terms] OR ""sudan""[All Fields] OR ""sudans""[All Fields] OR ""sudan s""[All Fields]) OR (""africa""[MeSH Terms] OR ""africa""[All Fields] OR ""africa s""[All Fields] OR ""africas""[All Fields]) OR (""ghana""[MeSH Terms] OR ""ghana""[All Fields] OR ""ghana s""[All Fields]) OR (""guinea""[MeSH Terms] OR ""guinea""[All Fields] OR ""guinea s""[All Fields] OR ""guineas""[All Fields]) OR (""guinea bissau""[MeSH Terms] OR ""guinea bissau""[All Fields] OR (""guinea""[All Fields] AND ""bissau""[All Fields]) OR ""guinea bissau""[All Fields]) OR (""kenya""[MeSH Terms] OR ""kenya""[All Fields] OR ""kenya s""[All Fields]) OR (""lesotho""[MeSH Terms] OR ""lesotho""[All Fields] OR ""lesotho s""[All Fields]) OR (""liberia""[MeSH Terms] OR ""liberia""[All Fields] OR ""liberi 1,039,609 time 20:11:28
**The Scopus Database search strategy**	
	("non communicable disease" OR "chronic conditions" OR "chronic health conditions" OR "chronic diseases" OR hypertension OR arteriosclerosis OR "cardiovascular disease" OR "blood pressure" OR diabetes OR "diabetes mellitus" OR "diabetes insipidus" OR "diabetes gestational" OR hyperglycaemia OR "blood glucose" OR "diabetes mellitus type 2" OR "type 2 diabetes" OR "diabetes mellitus type 1" OR neoplasms OR cancer OR "cervical cancer" OR "cancer of the cervix" OR malignancy OR tumor OR "prostate cancer" OR asthma OR "respiratory disorder" OR "bronchial asthma" OR "pulmonary disease chronic obstructive" OR "mental health" OR psychosocial OR psychotherapy OR epilepsy OR depression OR "mental disorders" OR schizophrenia OR "coronary artery disease" OR stroke OR "pulmonary hypertension") 165,537<br>AND (all "digital health" OR ehealth OR mhealth OR "mobile health" OR "digital technologie*" OR "digital intervention" OR telemedicine OR "tele medicine" OR telecare OR "tele consultation" OR text OR "video consult" OR android OR cellphone OR "cell phone" OR multimedia OR "personal digital assistant" OR sms OR software OR telecomm OR "information technology" OR internet OR iphone OR ipod OR web OR smartphone OR "mobile phone" OR "electronic health record" OR "personal health record" OR "computer based patient record" OR "health information system" OR "internet of medical things" OR robot OR "artificial intelligence" OR epharmacy 48,743<br>AND (all "task sharing" OR "task shifting" OR "community health worker" OR "non specialist health worker" OR "non physician health worker" OR "lay health worker" OR "village health worker" OR "lay counsellor" OR "lay worker" 23,197<br>AND PUBYEAR > 2014 AND PUBYEAR < 2025 AND (LIMIT-TO (AFFILCOUNTRY , "South Africa") OR LIMIT-TO (AFFILCOUNTRY , "Nigeria") OR LIMIT-TO (AFFILCOUNTRY , "Morocco") OR LIMIT-TO (AFFILCOUNTRY , "Ethiopia") OR LIMIT-TO (AFFILCOUNTRY , "Kenya") OR LIMIT-TO (AFFILCOUNTRY , "Uganda") OR LIMIT-TO (AFFILCOUNTRY , "Ghana") OR LIMIT-TO (AFFILCOUNTRY , "Tanzania") OR LIMIT-TO (AFFILCOUNTRY , "Malawi") OR LIMIT-TO (AFFILCOUNTRY , "Zimbabwe") OR LIMIT-TO (AFFILCOUNTRY , "Cameroon") OR LIMIT-TO (AFFILCOUNTRY , "Zambia") OR LIMIT-TO (AFFILCOUNTRY , "Senegal") OR LIMIT-TO (AFFILCOUNTRY , "Rwanda") OR LIMIT-TO (AFFILCOUNTRY , "Cote d'Ivoire") OR LIMIT-TO (AFFILCOUNTRY , "Burkina Faso") OR LIMIT-TO (AFFILCOUNTRY , "Mozambique") OR LIMIT-TO (

	AFFILCOUNTRY , "Congo") OR LIMIT-TO (AFFILCOUNTRY , "Botswana") OR LIMIT-TO (AFFILCOUNTRY , "Gambia") OR LIMIT-TO (AFFILCOUNTRY , "Benin") OR LIMIT-TO (AFFILCOUNTRY , "Democratic Republic Congo") OR LIMIT-TO (AFFILCOUNTRY , "Mali") OR LIMIT-TO (AFFILCOUNTRY , "Libya") OR LIMIT-TO (AFFILCOUNTRY , "Togo") OR LIMIT-TO (AFFILCOUNTRY , "Namibia") OR LIMIT-TO (AFFILCOUNTRY , "Sierra Leone") OR LIMIT-TO (AFFILCOUNTRY , "Somalia") OR LIMIT-TO (AFFILCOUNTRY , "Liberia") OR LIMIT-TO (AFFILCOUNTRY , "Guinea") OR LIMIT-TO (AFFILCOUNTRY , "Gabon") OR LIMIT-TO (AFFILCOUNTRY , "Lesotho") OR LIMIT-TO (AFFILCOUNTRY , "Swaziland")) AND (LIMIT-TO (EXACTKEYWORD , "Human")) AND (LIMIT-TO (DOCTYPE , "ar")) AND (LIMIT-TO (SUBJAREA , "NURS") OR LIMIT-TO (SUBJAREA , "MEDI") OR LIMIT-TO (SUBJAREA , "HEAL")) AND (LIMIT-TO (LANGUAGE , "English"))
**The Google Scholar search strategy**	
	https://scholar.google.com/scholar?as_q=digital+health+OR+ehealth+OR+e-health+OR+electronic+health+OR+digital+technologies+OR+digital+intervention+OR+electronic+care+OR+telemedicine+OR+telehealth+OR+telecare+OR+teleconsultation+OR+video+consultation+OR+text+message+OR+text+OR+mobile+health+OR+mhealth+OR+m-health+OR+android+OR+app+OR+audio+OR+cellphone+OR+computer+OR+mobile+OR+multi-media+OR+personal+digital+assistant+OR+SMS+OR+social+media+OR+software+OR+telecomm+OR+etherapy+OR+information+technology+OR+instant+message+OR+internet+OR+i-pad+OR+ipad+OR+iphone+OR+i-phone+OR+i-pod+OR+web+OR+smart+phone+OR+mobile+phone+OR+email+OR+e-mail+OR+electronic+health+record+OR+personal+health+record+OR+electronic+medical+record+OR+computer-based+patient+record+OR+health+information+system+OR+internet+of+medical+things+OR+robot+OR+artificial+intelligence+OR+epharmacy+AND+task+sharing+OR+distribution+of+tasks+OR+allocation+of+duties+OR+task+distribution+OR+sharing+of+tasks+OR+allocation+of+functions+OR+community+health+worker+OR+cooperative+behaviour+OR+task+shifting+OR+non-specialist+health+workers+OR+non-physician+health+workers+OR+distribution+of+duties+OR+task+shared+OR+sharing+tasks+OR+shifting+tasks+OR+lay+health+worker+OR+village+health+workers+OR+counsellor+AND+non-communicable+diseases+OR+chronic+conditions+OR+chronic+health+problem+OR+chronic+diseases+OR+hypertension+OR+arterioscelrosis+OR+cardiovascular+disease+OR+blood+pressure+OR+diabetes+OR+diabetes+mellitus+OR+diabetes+insipidus+OR+diabetes+gestational+OR+hyperglycaemia+OR+metabollic+syndrome+OR+hyperglycaemia+OR+blood+glucose+OR+diabetes+mellitus+Type+2+OR+diabetes+mellitus+type+1+OR+neoplasms+OR+cancer+OR+cervical+cancer+OR+cancer+of+the+cervix+OR+breast+cancer+OR+malignancy+OR+tumor+OR+prostate+cancer+OR+asthma+OR+respiratory+disorders+OR+bronchial+asthma+OR+pulmonary+disease+chronic+obstructive+OR+chronic+obstructive+pulmonary+disease+OR+mental+health+OR+psychosocial+support+OR+psychosocial+intervention+OR+psychotherapy+OR+eplilepsy+OR+depression+OR+depressive+symptoms+OR+depressions+OR+schizophrenia+OR+mental+disorders+AND+algeria+OR+africa+OR+angola+OR+benin+OR+botswana+OR+burkina+faso+OR+burundi+OR+carbo+verde+OR+cameroon+OR+cantral+africa+republic+OR+chad+OR+comoros+OR+democratic+republic+of+congo+OR+ivory+coast+OR+djibouti+OR+egypt+OR+equatorial+guinea+OR+eritrea+OR+eswatini+OR+ethiopia+OR+gabon+OR+gambia+OR+south+sudan+OR+sudan+OR+ghana+OR+guinea+OR+guinea+bissau+OR+kenya+OR+lesotho+OR+liberia+OR+libya+OR+madagascar+OR+malawi+OR+mali+OR+mauritania+OR+mauritius+OR+morocco+OR+mozambique+OR+namibia+OR+niger+OR+nigeria+OR+rwanda+OR+sao+tome+and+principe+OR+senegal+OR+seychelles+OR+sierra+leone+OR+somalia+OR+south+africa+OR+tanzania+OR+togo+OR+tunisia+OR+uganda+OR+zambia+OR+zimbabwe+
