# Supplementary material for: Use of digital health in task-sharing for prevention and management of non-communicable diseases in Africa: A scoping review
Source: PLOS Glob Public Health. 2026 Jul 24;6(7):e0006746. doi: 10.1371/journal.pgph.0006746 (PMC13399288; doi:10.1371/journal.pgph.0006746)
Supplement: S2 Appendix — (PDF) [file pgph.0006746.s003.pdf]

S2_Appendix

Use of Digital Health in Task-sharing for Prevention and Management of Non-Communicable Diseases in Africa: A Scoping Review

	Authors (Year)	Title of study	Objectives	Country	Setting	Study design/a	Sample size	Type of non-sp	NCD managed/	DHIs used	Study outcomes	Outcomes related to review
1	Catley, D., Puoane, T., Tsolekile, L., Resnicow, K., Fleming, K.K., Hurley, E.A., Smyth, J.M., Materia, F.T., Lambert, E.V., Vitolins, M.Z. and Levitt, N.S., 2022.	Evaluation of an adapted version of the Diabetes Prevention Program for low-and middle-income countries: A cluster randomized trial to evaluate "Lifestyle Africa" in South Africa	To examine the effectiveness of the adapted diabetes prevention program in LMIC	South Africa	Urban	Randomised control trial	494 participants: 215 in control arm and 223 in usual care arm (CHWs)	Community Health Workers	Type 2 Diabetes and CVD risks	Video based education sessions and mHealth	It was feasible for CHWs to deliver a lifestyle intervention using video based education	DHIs used in prevention of NCDs
2	Chibanda, D., Weiss, H.A., Verhey, R., Simms, V., Munjoma, R., Rusakaniko, S., Chingono, A., Munetsi, E., Bere, T., Manda, E. and Abas, M., 2016.	Effect of a primary care–based psychological intervention on symptoms of common mental disorders in Zimbabwe: a randomized clinical trial.	To assess the effectiveness of a culturally adapted psychosocial intervention on common mental health disorders	Zimbabwe	Urban	Randomised control trial	286 patients in the intervention group and 287 patients in the usual care group (24 CHWs	Lay health work	Mental health	mHealth	After 6 months patients in the intervention group had less symptoms of common mental health disorders	DHIs used in treatment of NCDs

3	Doukani, A., van Dalen, R., Valev, H., Njenga, A., Sera, F. and Chibanda, D., 2021.	A community health volunteer delivered problem-solving therapy mobile application based on the Friendship Bench 'Inuka Coaching'in Kenya: A pilot cohort study.	To assess effectiveness of a psychosocial intervention delivered through a mobile application by community health volunteers	Kenya	Kenya (type of setting not specified)	Cohort study	52	Community Health Workers	Mental health	mHealth	There was significant reduction in common mental health symptoms over a three month period	DHI use in providing a mental health intervention
4	Effah, K., Wormenor, M.C., Tekpor, E., Amuah, J.E., Atuguba, H.B., Mensah, E.N., Badzi, E.S., Danyo, S., Agyiri, D., Klutsey, G.B. and Akakpo, P.K., 2022.	Mobile colposcopy by trained nurses in a cervical cancer screening programme at Battor, Ghana.	To share experience of a cervical cancer screening program implemented by nurses in a low resource setting to mitigate staff shortage	Ghana	Predominantely rural with some urban catchment	Descriptive cross-sectional study	828 women.	Nurses	Cervical Cancer	mHealth	Nurses trained to provide cervical cancer screening can deliver the intervention	DHI use in screening
5	Mugisha, M., Ntakirutimana, I., Kayiranga, D., Muhirwa, A., Nkurunziza, E., Sibomana, E., Uwamahoro, A., Igihozo, T., Ndoli, A., Niyonshuti, E., Rwubaka, E., Tuyishime, I., Umutoniwase, E. M., and Mugisha, E. 2024	Muzima Mobile Application for Screening Hypertension and Diabetes: A User Experience of the App Among Community Health Workers in Rwanda.	To explore user experiences in using the Muzima mobile application among community health workers	Rwanda	Rwanda (nature of setting not specified)	Descriptive qualitative study	60 participants (10 Community health workers and 10 community environmental health workers)	Community Health Workers	Hypertension and diabetes	mHealth	The non-specialist HWs found that the Muzima app was useful to screen and amange Hypertenison and diabetes in the community, however the ease of use could be improved	Multiple uses of DHIs, challenges in using DHIs

6	Nelissen, H.E., Cremers, A.L., Okwor, T.J., Kool, S., van Leth, F., Brewster, L., Makinde, O., Gerrets, R., Hendriks, M.E., Schultsz, C. and Osibogun, A., 2018.	Pharmacy-based hypertension care employing mHealth in Lagos, Nigeria–a mixed methods feasibility study.	To assess the feasibility of a pharmacy based hypertension care model using mHealth	Nigeria	Urban	Mixed methods study	328 patients across 5 pharmacies	Pharmacists	Hypertension	mHealth	Patients accepted the screening for hypertension at pharmacies and there was a significant reduction of blood pressure among patients	DHI used for integration of services.
7	Niyibizi, J.B., Ntawuyirushintege, S., Nganabashaka, J.P., Umwali, G., Tumusiime, D., Ntaganda, E., Rulisa, S. and Bavuma, C.M., 2023.	Community health worker-led cardiovascular disease risk screening and referral for care and further management in rural and urban communities in Rwanda	To assess the ability of community health workers to screen and refer people at risk of CVD	Rwanda	Urban and rural	Action research (analytical cross-sectional)	498 patients in rural areas and 497 patients in urban areas.	Community health workers	Cardiovascular disease	mHealth (KoboCollect App)	There are agreement between cardiovascular risk assessment done by CHWs and that done by nurses in both rural and urban areas	DHI used for screening of patients for cardiovascular disease risk
8	O'Grady, M.A., Mootz, J., Suleman, A., Sweetland, A., Teodoro, E., Anube, A., Feliciano, P., Bezuidenhout, C., Dos Santos, P.F., Fumo, W. and Gouveia, L., 2022. .	Mobile technology and task shifting to improve access to alcohol treatment services in Mozambique	To describe feasibility, acceptability and appropriateness of a provider facing mHealth application, used under task shifting to screen for unhealthy alcohol use	Mozambiqu	Rural	Mixed methods study	15 participants in the qualitative arm of the study and 45 respondents in the quantitative arm.	Psychiatric technicians and primary care practitioners	Unhealthy alcohol use (mental health	mHealth	Non-specialist health workers found that the mHealth could be used for task shifting and its use was feasible and accpetable	DHI used in treatment and in screening for unhealthy alcohol use

9	Ojagbemi, A., Daley, S., Kola, L., Taylor Salisbury, T., Feeney, Y., Makhmud, A., Lempp, H., Thornicroft, G. and Gureje, O., 2022. .	Perception of providers on use of the WHO mental health Gap Action Programme-Intervention Guide (mhGAP-IG) electronic version and smartphone-based clinical guidance in Nigerian primary care settings	To assess perceptions of primary healthcare providers in the acceptability, feasibility and benefits of using the WHO mental health Gap Action Programme-Intervention and smart phones	Nigeria	Rural and u	Qualitative study	34 participants	Nurses, community health officers and community health extension officers.	Mental health	mHealth (Electronic version of the WHO mental health Gap Action programme intervention)	CHWs preffered using the mobile based application instead of paper-based guidance. mHealth apps could be used for other purposes either than clinical decision support and consultation	mHealth used in the treatment of mental health conditions
10	Paddick, S.M., Yoseph, M., Gray, W.K., Andrea, D., Barber, R., Colgan, A., Dotchin, C., Urasa, S., Kissima, J., Haule, I. and Kisoli, A., 2021.	Effectiveness of app-based cognitive screening for dementia by lay health workers in low resource settings. A validation and feasibility study in rural Tanzania.	To assess the diagnostic screening accuracy of an mHealth app for dementia by non-specialist HWs	Tanzania	Rural	Cross-sectional analytical study	3011	Community Health Workers	Mental health	mHealth	The mHealth application had good sensitivity but poor specificity when used by non-specialist HWs to screen for dementia	mHealth used in screening for NCDs
11	Jacobs, Y., Myers, B., Van Der Westhuizen, C., Brooke-Sumner, C. and Sorsdahl, K., 2021.	Task sharing or task dumping: counsellors experiences of delivering a psychosocial intervention for mental health problems in South Africa	To explore the experiences of facility-based counsellors in the provision of counselling for chronic conditions	South Africa	Nature of setting unspecified	Qualitative stu	34	Facility based counsellors	Mental health	mHealth	Training improved facility based counsellors confidence and this was supported by structured supervision	Telephonic supervision of non-specialist health workers

12	Selohilwe, O., Fairall, L., Bhana, A., Kathree, T., Zani, B., Folb, N., Lund, C., Thornicroft, G. and Petersen, I., 2023.	Challenges and opportunities for implementation and dissemination of a task-sharing counselling intervention for depression at primary health care level in South Africa.	To describe the multilevel factors influencing implementation and dissemination of a lay counselling service in South Africa	South Africa	Nature of s	Qualitative stu	86 participants	Lay health counsellors	Mental health	mHealth	Facilitators of task-sharing included supervision, person centred approach and integration of services. Barriers included lack of space, high counsellor turnover	Telephonic supervision of non-specialist health workers
13	Triplett, N.S., Johnson, C., Kiche, S., Dastrup, K., Nguyen, J., Daniels, A., Mbwayo, A., Amanya, C., Munson, S., Collins, P.Y. and Weiner, B.J., 2023.	Understanding lay counselor perspectives on mobile phone supervision in Kenya: Qualitative study.	To describe the multilevel factors influencing implementation and dissemination of a lay counselling service in South Africa	Kenya	Nature of s	Qualitative stu	27 participants	Lay counselllors	Mental health	mHealth	Mobile phone use included sceduling appointments, providing clinical updates and supporting research procedures	Telephonic supervision of non-specialist health workers
14	Tsai, A.C., Tomlinson, M., Dewing, S., Le Roux, I.M., Harwood, J.M., Chopra, M. and Rotheram-Borus, M.J., 2014.	Antenatal depression case finding by community health workers in South Africa: feasibility of a mobile phone application.	To assess the extent to which CHWs could be trained to screen for antenatal depression using mobile phones	South Africa	Low socio-	Cross-sectional analytical study	Study 1 had 1144 participants, study 2 had 361 participants	Community Health Workers	Mental health	mHealth	It was feasible for CHWs to screen for depression using an mHealth app	mHealth in the screening for antenatal depression

15	Vedanthan, R., Kamano, J.H., DeLong, A.K., Naanyu, V., Binanay, C.A., Bloomfield, G.S., Chrysanthopoulou, S.A., Finkelstein, E.A., Hogan, J.W., Horowitz, C.R. and Inui, T.S., 2019.	Community health workers improve linkage to hypertension care in Western Kenya.	To determine whether community health workers equipped with smart phone technologies and behaviour change communication can increase access to a hypertension care program	Kenya	Rural	Randomised c	1460 individuals 491 usual care, 500 paper-based, 469 smartphone.	Community Health Volunteers	Hypertension	mHealth	Participants who used smart phones had slightly lower levells of hypertension	DHI used in prevention of NCDs
