# Supplementary material for: Use of digital health in task-sharing for prevention and management of non-communicable diseases in Africa: A scoping review
Source: PLOS Glob Public Health. 2026 Jul 24;6(7):e0006746. doi: 10.1371/journal.pgph.0006746 (PMC13399288; doi:10.1371/journal.pgph.0006746)
Supplement: Attachment — Submitted filename: Point by Point Response to reviewers_AGT.docx [file pgph.0006746.s006.docx]

**6 April 2026**

**Response to Reviewers**

**Author Response Letter: PLOS Global Public Health**

**Dear Academic Editor, Zulkarnain Jaafar**

I am writing on behalf of all authors to submit the revised manuscript on “Use of Digital Health in Task-sharing for Prevention and Management of Non-Communicable Diseases in Africa: A Scoping Review”. We are grateful for the opportunity to respond to the reviewers’ comments and have prepared a point-by-point response below. A clean manuscript and a tracked change manuscript are also submitted per the Journal’s requirements.

**Reviewer 1**

**Comment:** Line 64 "... other conditions, including mental health instead of "... other conditions, such as mental health" (minor)

**Response**: The sentence has been changed to read… other conditions, including mental health conditions” page 3, line 66 (tracked changes manuscript).

**Comment**: Line 100: It might be good to mention if the protocol was registered in OSF before the initiation of the study.

**Response:** We have added that the protocol was registered before the start of the study. Page 5, lines 111-112

**Comment:** Line 115: "using AI to process clinical and genetic data" (not sure if this definition is comprehensive enough as it highlights only AI here. Instead use the word 'technologies' instead of AI as it might be a generic representation.

**Response:** We have corrected the definition of digital health, and the sentence now shows that the word “technologies” instead of “AI” are used to process genetic and clinical data. Line 118 page 5.

**Comment:** Line 130: It might be helpful to mention the exact date of the search instead of August 2025. If anyone wants to do another search the date will be helpful to know.

**Response:** We have included the exact dates of the search. Page 6, lines 133 to 134

**Comment:** Line 139: Not sure if we are assessing risk of bias here in this review. Please modify accordingly.

**Response:** The risk of bias has been deleted. Line 143, page 6.

**Comment:** Line 141: independently reviewed (does it include both title and abstract screening, and full text screening?) thought it might be good to clarify.
**Response:** We have clarified that both title and abstract screening were done independently. Page 6, lines 146 to 147.

**Comment:** line 143: consulting the research team (it might be helpful to provide more clarity here on who resolved the discrepancies)
**Response**: We have clarified that disputes were resolved by consulting the co-authors: AGT, SAM and RJ, Page 6, line 148.

**Results:**
**Comment**: Please verify the number of articles 4857 - the numbers do not seem to add up.

**Response:** We have revised lines 167-169to align with the PRISMA flow diagram.

**Comment:** Table 1: Consistent use of terminology: eg., SMS and SMSs. Also, please provide abbreviation for SMS

**Response:** We have edited the term “SMS” to ensure consistency with the sentence structure and provided the abbreviation (page 13, line 195 and page 21, line 318).

**Comment:** Line 197: " while pharmacists, psychiatric technicians and primary care practitioners" is preferably. suggest you to remove the 'and'

**Response:** The term “and” has been deleted from the above-mentioned sentence, page 14, line 203.

**Comment:** Line 200: Consistent use of percentage will be helpful. Eight studies are provided with the % and missing in the first line with seven studies.

**Response:** The percentage of the seven studies has been included. Page 14, line 206.

**Comment:** Line 207: suggestion: "mHealth was the commonly used digital technology in the included studies" Line 207: Please rephrase the line with two dashes " - which described the use of mHealth - "

**Response**: The sentence has been rephrased as suggested. Page 14, line 214

**Comment:** Line 208: Not sure what the word 'this' refers here.

**Response:** The word “this” refers to smart mobile phone applications; the sentence has been revised. Page 14, line 217.

**Comment**: Line 348: 'previous scoping review' instead of previous systematic review.

**Response**: The sentence has been corrected to previous scoping review. Page, 23, line 356

**Comment:** Conclusion: Might be good to mention that there are cost issues are there as well. Would be interesting to note if any of the studies included made any mention of the costs involved with the technology and implementation.

**Response:** We have included the cost implications for scaling up digital health technologies in the African countries(page 26, lines 430-431). The studies reviewed did not mention the costs involved in the implemented technologies.

**Reviewer #2:**

I would like to thank the authors, who integrated all of my suggestions in their article, which in my opinion is now ready to be published.

**Response: We thank the reviewers for the kind comment.**

Thank you again for your feedback and for considering our revised manuscript. Please do not hesitate to contact me if you have any further questions.

**Kind Regards**

**Nongiwe Linette Mhlanga**
