# Supplementary material for: Use of digital health in task-sharing for prevention and management of non-communicable diseases in Africa: A scoping review
Source: PLOS Glob Public Health. 2026 Jul 24;6(7):e0006746. doi: 10.1371/journal.pgph.0006746 (PMC13399288; doi:10.1371/journal.pgph.0006746)
Supplement: Attachment — Submitted filename: Point by Point Response to reviewers.docx [file pgph.0006746.s005.docx]

**Manuscript title: Use of Digital Health in Task-sharing for Prevention and Management of Non-Communicable Diseases in Africa: A Scoping Review**

**Author Response Letter: PLOS Global Public Health**

**Dear Academic Editor, Zulkarnain Jaafar**

I am writing on behalf of all authors to submit the revised manuscript on “Use of Digital Health in Task-sharing for Prevention and Management of Non-Communicable Diseases in Africa: A Scoping Review”. We are grateful for the opportunity to respond to the reviewers’ comments and have prepared a point-by-point response below. A clean manuscript, a tracked change manuscript, revised figures, and a supplementary file (S1) are also submitted per the Journal’s requirements.

**Editor’s comments**

**Comment:** We have noticed that you have uploaded Supporting Information files, but you have not included a list of legends. Please add a full list of legends for your Supporting Information files before or after the references list.

**Response:** We have included the full list of legends of supporting information before the references on page 25, lines 435 to 437

**Reviewers’ comments**

**Reviewer 1**

Dear Authors: Well done with the submission of this manuscript to this journal. Please find comments to help improve the manuscript for better understanding and clarity.

**Title:**
**Comment:** Easy to understand title, may I suggest that you add your significant findings in the title to make it sound better?

**Response:** Thank you for your suggestion. We appreciate the reviewer’s comment that the title is clear and easily understandable. In light of this, and based on our collective agreement, we have decided to retain the original title. Including additional outcomes in the title could overshadow some key findings and make it longer and less clear for the reader.
**Abstract:**
**Comment:** Line 2 says: “…. And improve the quality of care provided by health workers”. I am not sure if this statement is factually, right? Suggest to modify it based on appropriate evidence.

**Response:** We have revised the sentence, which now reads that digital health used in task-sharing is useful “to address workforce shortage and improve the delivery of health services”. Please refer Page 2, lines 35 and 36 of the revised manuscript (Track Changed Manuscript)

**Comment:** Suggest to include the number of articles along with the percentage. “.. and in 40% of studies” the percentage without the baseline value is misleading.
The abstract is written in a simple and easy to understand manner. Well done!
OSF protocol registration: the second link provided doesn’t seem to work, please check and mend the link. Also, I don’t seem to find the methods used by the authors for this review in the protocol in the first link. Please check and verify.

**Response:** We have included the baseline values for all percentages included in the abstract. Page 2, lines 45 and 46. Concerning the links for the protocol, thank you for also pointing this out. We have updated the protocol's contents and included the methods section. Page 2, line 55.

**Introduction:**
**Comment:** Line 100 to 102: “Evidence suggests that non-physician health workers (NPHWs)—such as community 100 health workers (CHWs)—can deliver basic health services that have typically been delivered by 101 professional health workers” – Can the authors add reference for this statement please, this is a very important statement, it might be better for this statement to have its own reference.

**Response:** We have included two references which indicate that CHWs can deliver basic health services. Page 4, line 85

**Comment:** Line 108 -109. I am unable to understand the significance of this statement “… intelligent processing of genetic and clinical data” might be helpful to explain the significance.

**Response:** We have revised the sentence to enhance clarity. The statement is part of the definition of Digital health, which illustrates that digital health incorporates the use of artificial intelligence (AI) to process patient data for improving patient care. Page 4, lines 91-92.

**Comment:** Line 91, 109: Minor – It might be better to add the full form before using the abbreviation, World Health Organization (WHO).

**Response:** WHO has been written out in full for the first time. Page 3, line 74

**Comment:** Line 110: minor – LMIC abbreviation

**Response:** We have written out LMIC in full. Page 4, line 94

**Comment:** Line 114-115: Please add appropriate reference for this statement “While the use of digital health demonstrates promise in supporting task-sharing for the prevention and management of NCDs elsewhere”

**Response:** We agree with the comment and have added a reference for the statement. Page 4, line 98

**Material and methods:**
**Comment:** Line 134 to 135: seems really out of place. “Digital health use was defined as the use of 134 technologies to provide a health service [11].” Suggestion: please add a better version for definition of digital health.

**Response:** We have revised the definition of digital health, which is consistent with the definition in the introduction. Page 5, lines 113-115

**Comment:** Line 147: It might be good to acknowledge the help of the librarian, and also mention if they assisted in any peer review of the search strategy (if done – it might be an additional strong point for methodology)

**Response:** We have acknowledged the assistance of the librarian, Mkhululi Dimane, who assisted with article retrieval. Page 24, Lines 439-440

**Comment:** Line 148: Not sure if they meant if the database search strategy was developed or the database searching was ‘performed on’. Kindly clarify.

**Response:** We thank the reviewer for the suggestion. The database search strategy was developed on the 7^th^ of April 2025, and we searched articles from April to August; however, the search strategy was developed in April, clarity has been provided. Page 5, line 129-130.

**Comment**: Line 153: Is there any reason for searching from 2014? Also, it might be good to include the date of search.

**Response:** We have clarified that the searching was done until August 2025, Page 5, lines 129-130. We have also clarified that we included studies from 2014, to enable a duration of more than 10 years to analyse any changes made in technologies. Page 6, lines 133-134.

**Comment:** Line 156: Can the authors please clarify which grey literature search was performed, and if they have used Google Scholar, how did they arrive at only 250 records?

**Response:** We have clarified that grey literature was searched from institutional websites such as the WHO and higher education institutions. Page 6, lines 136-139. We have also clarified that 250 records were identified from Google Scholar, as the first 25 pages were searched, and each page contained 10 articles. Page 5, lines 125 to 126.

**Comment:** Assessment of risk of bias: I am not sure how this adds to the paper? And if it's really needed for a scoping review.

**Response:** We have removed the risk of bias assessment.

**Comment:** Please check if the references 20 to 22 are quoted correctly.

**Response**: The references for Moola (2022) and Lockwood (2015) have been removed as the assessment of risk of bias has been deleted.

**Results:**
**Comment:** I am not sure if reference 24 should cite about PRISMA or PRISMA ScR. Please check.

**Response:** The figure was adapted from the PRISMA flow chart by Page et al. which is the PRISMA.

**Results:**
**Comment**: Please add numbers along with the % for 40% and 30% (line 202)

**Response:** The numbers along with the percentages have been included. Page 8, lines 173 and 174.

**Comment:** Regarding Table 1: Did the authors have any classification system they used to classify NCDs? It might be helpful for the readers to know how they identified the conditions. And if they used it as part of their search strategy, too?

**Response**: The definition of non-communicable diseases in the introduction has been added. This definition was also applied in the development of the search strategy. Page 3, lines 62-64

**Comment**: Suggest explanations of all abbreviations used in the table as a footnote, please.
Table 2 – Typo for author ‘Vedanthan’ [Ref 38]
**Response:** The footnotes that provide full explanations of all abbreviations have been included**.** Page 11, lines 188-189, Page 18, lines 312 to 313. We have corrected the spelling error for “Vedanthan” in Table 2. Page 18.

**Discussion:**
**Comment:** Would recommend consistent use of terminology “Consistent with this, our study found neutral results for digital health in NCD prevention and positive outcomes for treatment, albeit only four studies were included in the review.” (Digital health and mHealth).

**Response:** We have revised the sentence and used consistent terminology throughout the discussion. Page 21, lines 386-388

**Comment:** Figure 1: It might be good to provide the list of studies as a separate appendix rather than listing the titles in the flow chart.

**Response:** S1 Appendix provides the full list of articles.

**Comment:** PRISMA ScR checklist – please check if the page number for the conclusion is accurate.

**Response:** We have checked the page number of the conclusion and corrected it. SI Prisma checklist page 2,

**Comment:** 22 Funding – please add a statement about funding and cite the page number, even if no funding was received.

**Response:** The funding information has been included in the SI PRISMA Checklist. SI Prisma checklist page 2

**Comment:** S2 (Protocol): I am not sure how this might work, adding a protocol in a public domain might be the better way than to provide a protocol during peer review. The attached protocol says 2015 to 2025 for the articles search, and the review paper mentions 2014, there is a discrepancy. Please explain.
**Response:** We have included the protocol in a public domain that includes the methods section. The link is <https://doi.org/10.17605/OSF.IO/Q2XF9>. The discrepancy of including articles from 2014, rather than 2015, as initially planned, was because there was a paucity of literature from 2015 onwards; therefore, we broadened the search to include an additional year.

**Comment**: Zip file S1 – Search strategy, might have been helpful to add the search data with the number of hits on that day.

**Response:** The search data indicating the number of hits on the day and the times we searched has been included. SI Appendix, pages 1,2,4,6, and 7.

**Comment:** S2 – well done! Looks very comprehensive

**Response:** Thank you

**Comment: S3**: Not sure if this is needed, as they have not used it to explain their findings. Doesn’t really add any value to this review at the moment.

**Response:** We thank the reviewer for the suggestion. The supplementary file has been removed.

**REVIEWER 2**

**Reviewer #2:** The article addresses a very interesting topic and is scientifically solid. It has been a pleasure to read. Below are some minor suggestions for the authors to consider:

**Comment:** I am not a native English speaker, but at times the language sounds slightly unusual. It may be helpful to have a native speaker review the manuscript for a final linguistic check.

**Response:** We thank the reviewer for the kind note and helpful advice. The manuscript has been edited in detail by the research team to enhance clarity. However, as the edits were significant, we prefer not to include them in the revised manuscript.

**Comment:** For completeness, I suggest adding a very brief definition of non-communicable diseases at the beginning.

**Response:** The definition of non-communicable diseases in the introduction has been added. Page 3, lines 62-64.

**Comment**: I am not sure if this is an issue on my end, but Figure 1 appears to have very low resolution. Improving its quality would make it more readable. I would also have appreciated a second figure summarizing the general results (e.g., which technologies are used for which purposes), although this is just a suggestion.

**Response:** We have redone the image for Figure 1 using the link provided by the journal <https://ngplosjournals.pagemajik.ai/artanalysis> (Appendix reattached). Thank you for your suggestion. We have also added a figure (SI, Fig. 2) that summarises the technologies used and their purposes in NCD management and prevention. A paragraph explaining the figure is included on pages 15 & 16, lines 283-288

**Comment**: The current division into chapters feels a bit unusual. A more standard structure, perhaps using main sections (e.g., Methodology) and sub-sections (e.g., Research Questions), might make the paper clearer. Referring to the journal’s guidelines could also be helpful.

**Response:** As per the journal’s guidelines, we have revised the 'Methods and Materials' section and removed the subsections. In the results section, we have maintained subsections aligned with the research questions to facilitate a logical flow from the methods section.

Thank you again for your feedback and for considering our revised manuscript. Please do not hesitate to contact me if you have any further questions.

**Kind Regards**

**Nongiwe Linette Mhlanga**
