# Supplementary material for: Use of digital health in task-sharing for prevention and management of non-communicable diseases in Africa: A scoping review
Source: PLOS Glob Public Health. 2026 Jul 24;6(7):e0006746. doi: 10.1371/journal.pgph.0006746 (PMC13399288; doi:10.1371/journal.pgph.0006746)
Supplement: Attachment — Submitted filename: Point_by_Point_Response_to_reviewers_auresp_4.docx [file pgph.0006746.s008.docx]

**5 June 2026**

**Response to Reviewers**

**Author Response Letter: PLOS Global Public Health**

**Dear Academic Editor, Zulkarnain Jaafar**

I am writing on behalf of all authors to submit the revised manuscript on “Use of Digital Health in Task-sharing for Prevention and Management of Non-Communicable Diseases in Africa: A Scoping Review”. We are grateful for the opportunity to respond to the reviewers’ comments and have prepared a point-by-point response below. A clean manuscript and a tracked change manuscript are also submitted per the Journal’s requirements.

**Reviewer 1**

**Comment:** Great job in addressing all the comments. I have only one comment related to the PRISMA flowchart. The numbers still do not add up. Records identified = 4857. Records removed = 3580. So, the number of records screened must be 1277 and NOT 1295. This is what I meant by the numbers not adding up.

**Response:** We have revised the PRISMA flowchart and corrected the number of screened records to 1277, as indicated (Supplementary File figures). We have also made corrections in the manuscript on page 8, lines 167 and 168.

Thank you again for your feedback and for considering our revised manuscript. Please do not hesitate to contact me if you have any further questions.

**Kind Regards**

**Nongiwe Linette Mhlanga**
