# Supplementary material for: Use of digital health in task-sharing for prevention and management of non-communicable diseases in Africa: A scoping review
Source: PLOS Glob Public Health. 2026 Jul 24;6(7):e0006746. doi: 10.1371/journal.pgph.0006746 (PMC13399288; doi:10.1371/journal.pgph.0006746)
Supplement: Peer review history

# Peer review: Decision Letter 0

**DOI:** 10.1371/journal.pgph.0006746.r001

**Authors:** Zulkarnain Jaafar

14 Dec 2025

PGPH-D-25-02956

Use of Digital Health in Task-sharing for Prevention and Management of Non-Communicable Diseases in Africa: A Scoping Review

PLOS Global Public Health

Dear Dr. Mhlanga,

Thank you for submitting your manuscript to PLOS Global Public Health. After careful consideration, we feel that it has merit but does not fully meet PLOS Global Public Health’s publication criteria as it currently stands. Therefore, we invite you to submit a revised version of the manuscript that addresses the points raised during the review process.

==============================

EDITOR: Dear Author, please attend to all comments provided by the reviewers and make necessary corrections.

==============================

Please submit your revised manuscript by 11th March 2026. If you will need more time than this to complete your revisions, please reply to this message or contact the journal office at globalpubhealth@plos.org. When you're ready to submit your revision, log on to https://www.editorialmanager.com/pgph/ and select the 'Submissions Needing Revision' folder to locate your manuscript file.

Please include the following items when submitting your revised manuscript:

- A letter that responds to each point raised by the editor and reviewer(s). You should upload this letter as a separate file labeled 'Response to Reviewers'.
- A marked-up copy of your manuscript that highlights changes made to the original version. You should upload this as a separate file labeled 'Revised Manuscript with Track Changes'.
- An unmarked version of your revised paper without tracked changes. You should upload this as a separate file labeled 'Manuscript'.

Guidelines for resubmitting your figure files are available below the reviewer comments at the end of this letter.

We look forward to receiving your revised manuscript.

Kind regards,

Zulkarnain Jaafar

Academic Editor

PLOS Global Public Health

Journal Requirements:

1. If the reviewer comments include a recommendation to cite specific previously published works, please review and evaluate these publications to determine whether they are relevant and should be cited. There is no requirement to cite these works unless the editor has indicated otherwise.

Additional Editor Comments (if provided):

[Note: HTML markup is below. Please do not edit.]

Reviewers' comments:

Reviewer's Responses to Questions

**Comments to the Author**

1. Does this manuscript meet PLOS Global Public Health’s publication criteria? Is the manuscript technically sound, and do the data support the conclusions? The manuscript must describe methodologically and ethically rigorous research with conclusions that are appropriately drawn based on the data presented.

Reviewer #1: Yes

Reviewer #2: Yes

**********

2. Has the statistical analysis been performed appropriately and rigorously?

Reviewer #1: N/A

Reviewer #2: N/A

**********

3. Have the authors made all data underlying the findings in their manuscript fully available (please refer to the Data Availability Statement at the start of the manuscript PDF file)?

The PLOS Data policy requires authors to make all data underlying the findings described in their manuscript fully available without restriction, with rare exception. The data should be provided as part of the manuscript or its supporting information, or deposited to a public repository. For example, in addition to summary statistics, the data points behind means, medians and variance measures should be available. If there are restrictions on publicly sharing data—e.g. participant privacy or use of data from a third party—those must be specified.

Reviewer #1: Yes

Reviewer #2: Yes

**********

4. Is the manuscript presented in an intelligible fashion and written in standard English?

PLOS Global Public Health does not copyedit accepted manuscripts, so the language in submitted articles must be clear, correct, and unambiguous. Any typographical or grammatical errors should be corrected at revision, so please note any specific errors here.

Reviewer #1: Yes

Reviewer #2: Yes

**********

5. Review Comments to the Author

Please use the space provided to explain your answers to the questions above. You may also include additional comments for the author, including concerns about dual publication, research ethics, or publication ethics. (Please upload your review as an attachment if it exceeds 20,000 characters)

Reviewer #1: Dear Authors,

Well done with the submission of this manuscript to this journal. Please find comments to help improve the manuscript for better understanding and clarity.

Title: Easy to understand title, may I suggest if you could add your significant findings in the title to make it sound better?

Abstract:

Line 2 says: “…. And improve the quality of care provided by health workers”. I am not sure if this statement is factually, right? Suggest to modify it based on appropriate evidence.

Suggest to include the number of articles along with the percentage. “.. and in 40% of studies” the percentage without the baseline value is misleading.

The abstract is written in a simple and easy to understand manner. Well done!

OSF protocol registration: the second link provided doesn’t seem to work, please check and mend the link. Also, I don’t seem to find the methods used by the authors for this review in the protocol in the first link. Please check and verify.

Introduction:

Line 100 to 102: “Evidence suggests that non-physician health workers (NPHWs)—such as community 100 health workers (CHWs)—can deliver basic health services that have typically been delivered by 101 professional health workers” – Can the authors add reference for this statement please, this is a very important statement, it might be better for this statement to have its own reference.

Line 108 -109. I am unable to understand the significance of this statement “… intelligent processing of genetic and clinical data” might be helpful to explain the significance.

Line 91, 109: Minor – It might be better to add the full form before using the abbreviation, World Health Organization (WHO).

Line 110: minor – LMIC abbreviation

Line 114-115: Please add appropriate reference for this statement “While the use of digital health demonstrates promise in supporting task-sharing for the prevention and management of NCDs elsewhere”

Material and methods:

Line 134 to 135: seems really out of place. “Digital health use was defined as the use of 134 technologies to provide a health service [11].” Suggestion: please add a better version for definition of digital health.

Line 147: It might be good to acknowledge the help of the librarian, and also mention if they assisted in any peer review of the search strategy (if done – it might be an additional strong point for methodology)

Line 148: Not sure if they meant if the database search strategy was developed or the data base searching was ‘performed on’. Kindly clarify.

Line 153: Is there any reason for searching from 2014? Also, it might be good to include the date of search.

Line 156: Can the authors please clarify which grey literature search was performed, and if they have used Google Scholar, how did they arrive at only 250 records?

Assessment of risk of bias: I am not sure how this adds to the paper? And if its really needed for a scoping review.

Please check if the references 20 to 22 are quoted correctly.

Results:

I am not sure if reference 24 should cite about PRISMA or PRISMA ScR. Please check.

Results:

Please add numbers along with the % for 40% and 30% (line 202)

Regarding Table 1: Did the authors have any classification system they used to classify NCDs? It might be helpful for the readers to know how they identified the conditions. And if they used it as part of their search strategy too?

Suggest explanations of all abbreviations used in the table as a foot note please.

Table 2 – Typo for author ‘Vedanthan’ [Ref 38]

Discussion:

Would recommend consistent use of terminology “Consistent with this, our study found neutral results for digital health in NCD prevention and positive outcomes for treatment, albeit only four studies were included in the review.” (Digital health and mHealth)

Figure 1: It might be good to provide the list of studies as a separate appendix rather than listing the titles in the flow chart.

PRISMA ScR checklist – please check if the page number for conclusion is accurate.

22 Funding – please add a statement about funding and cite the page number, even if not funding was received.

S2 (Protocol): I am not sure how this might work, adding a protocol in a public domain might be the better way than to provide a protocol during peer review. The attached protocol says 2015 to 2025 for the articles search, and the review paper mentions 2014, there is a discrepancy. Please explain.

Zip file

S1 – Search strategy, might have been helpful to add the search data with the number of hits on that day.

S2 – well done! Looks very comprehensive

S3: Not sure if this is needed, as they have not used it to explain their findings. Doesn’t really add any value to this review at the moment.

Reviewer #2: The article addresses a very interesting topic and is scientifically solid. It has been a pleasure to read. Below are some minor suggestions for the authors to consider:

I am not a native English speaker, but at times the language sounds slightly unusual. It may be helpful to have a native speaker review the manuscript for a final linguistic check.

For completeness, I suggest adding a very brief definition of non-communicable diseases at the beginning.

I am not sure if this is an issue on my end, but Figure 1 appears to have very low resolution. Improving its quality would make it more readable. I would also have appreciated a second figure summarizing the general results (e.g., which technologies are used for which purposes), although this is just a suggestion.

The current division into chapters feels a bit unusual. A more standard structure, perhaps using main sections (e.g., Methodology) and sub-sections (e.g., Research Questions), might make the paper clearer. Referring to the journal’s guidelines could also be helpful.

**********

6. PLOS authors have the option to publish the peer review history of their article (what does this mean?). If published, this will include your full peer review and any attached files.

**Do you want your identity to be public for this peer review?**  If you choose “no”, your identity will remain anonymous but your review may still be made public.

For information about this choice, including consent withdrawal, please see our Privacy Policy.

Reviewer #1: No

Reviewer #2: No

**********

[NOTE: If reviewer comments were submitted as an attachment file, they will be attached to this email and accessible via the submission site. Please log into your account, locate the manuscript record, and check for the action link "View Attachments". If this link does not appear, there are no attachment files.]

-->Figure Resubmissions:

While revising your submission, we strongly recommend that you use PLOS’s NAAS tool (https://ngplosjournals.pagemajik.ai/artanalysis) to test your figure files. NAAS can convert your figure files to the TIFF file type and meet basic requirements (such as print size, resolution), or provide you with a report on issues that do not meet our requirements and that NAAS cannot fix.

After uploading your figures to PLOS’s NAAS tool - https://ngplosjournals.pagemajik.ai/artanalysis, NAAS will process the files provided and display the results in the "Uploaded Files" section of the page as the processing is complete. If the uploaded figures meet our requirements (or NAAS is able to fix the files to meet our requirements), the figure will be marked as "fixed" above. If NAAS is unable to fix the files, a red "failed" label will appear above. When NAAS has confirmed that the figure files meet our requirements, please download the file via the download option, and include these NAAS processed figure files when submitting your revised manuscript.

# Author comment: Author response to Decision Letter 1

**DOI:** 10.1371/journal.pgph.0006746.r002

23 Feb 2026

Attachment Submitted filename: Point by Point Response to reviewers.docx

# Peer review: Decision Letter 1

**DOI:** 10.1371/journal.pgph.0006746.r003

**Authors:** Zulkarnain Jaafar

23 Mar 2026

PGPH-D-25-02956R1

Use of Digital Health in Task-sharing for Prevention and Management of Non-Communicable Diseases in Africa: A Scoping Review

PLOS Global Public Health

Dear Dr. Mhlanga,

Thank you for submitting your manuscript to PLOS Global Public Health. After careful consideration, we feel that it has merit but does not fully meet PLOS Global Public Health’s publication criteria as it currently stands. Therefore, we invite you to submit a revised version of the manuscript that addresses the points raised during the review process.

==============================

EDITOR: Dear Author, this manuscript still requiring a minor revision to be made.

==============================

Please submit your revised manuscript by 19th April 2026. If you will need more time than this to complete your revisions, please reply to this message or contact the journal office at globalpubhealth@plos.org. When you're ready to submit your revision, log on to https://www.editorialmanager.com/pgph/ and select the 'Submissions Needing Revision' folder to locate your manuscript file.

Please include the following items when submitting your revised manuscript:

- A letter that responds to each point raised by the editor and reviewer(s). You should upload this letter as a separate file labeled 'Response to Reviewers'.
- A marked-up copy of your manuscript that highlights changes made to the original version. You should upload this as a separate file labeled 'Revised Manuscript with Track Changes'.
- An unmarked version of your revised paper without tracked changes. You should upload this as a separate file labeled 'Manuscript'.

Guidelines for resubmitting your figure files are available below the reviewer comments at the end of this letter.

We look forward to receiving your revised manuscript.

Kind regards,

Zulkarnain Jaafar

Academic Editor

PLOS Global Public Health

Journal Requirements:

If the reviewer comments include a recommendation to cite specific previously published works, please review and evaluate these publications to determine whether they are relevant and should be cited. There is no requirement to cite these works unless the editor has indicated otherwise.

Please review your reference list to ensure that it is complete and correct. If you have cited papers that have been retracted, please include the rationale for doing so in the manuscript text, or remove these references and replace them with relevant current references. Any changes to the reference list should be mentioned in the rebuttal letter that accompanies your revised manuscript. If you need to cite a retracted article, indicate the article’s retracted status in the References list and also include a citation and full reference for the retraction notice.

Additional Editor Comments (if provided):

[Note: HTML markup is below. Please do not edit.]

Reviewers' comments:

Reviewer's Responses to Questions

**Comments to the Author**

1. If the authors have adequately addressed your comments raised in a previous round of review and you feel that this manuscript is now acceptable for publication, you may indicate that here to bypass the “Comments to the Author” section, enter your conflict of interest statement in the “Confidential to Editor” section, and submit your "Accept" recommendation.

Reviewer #1: (No Response)

Reviewer #2: All comments have been addressed

**********

2. Does this manuscript meet PLOS Global Public Health’s publication criteria? Is the manuscript technically sound, and do the data support the conclusions? The manuscript must describe methodologically and ethically rigorous research with conclusions that are appropriately drawn based on the data presented.

Reviewer #1: Yes

Reviewer #2: Yes

**********

3. Has the statistical analysis been performed appropriately and rigorously?

Reviewer #1: N/A

Reviewer #2: Yes

**********

4. Have the authors made all data underlying the findings in their manuscript fully available (please refer to the Data Availability Statement at the start of the manuscript PDF file)?

The PLOS Data policy requires authors to make all data underlying the findings described in their manuscript fully available without restriction, with rare exception. The data should be provided as part of the manuscript or its supporting information, or deposited to a public repository. For example, in addition to summary statistics, the data points behind means, medians and variance measures should be available. If there are restrictions on publicly sharing data—e.g. participant privacy or use of data from a third party—those must be specified.

Reviewer #1: Yes

Reviewer #2: Yes

**********

5. Is the manuscript presented in an intelligible fashion and written in standard English?

PLOS Global Public Health does not copyedit accepted manuscripts, so the language in submitted articles must be clear, correct, and unambiguous. Any typographical or grammatical errors should be corrected at revision, so please note any specific errors here.

Reviewer #1: Yes

Reviewer #2: Yes

**********

6. Review Comments to the Author

Please use the space provided to explain your answers to the questions above. You may also include additional comments for the author, including concerns about dual publication, research ethics, or publication ethics. (Please upload your review as an attachment if it exceeds 20,000 characters)

Reviewer #1: Dear Authors,

Thank you for addressing the earlier comments. A few suggestions to help improve the manuscript.

Line 64 "... other conditions, including mental health instead of "... other conditions, such as mental health" (minor)

Line 100: It might be good to mention if the protocol was registered in OSF before the initiation of the study.

Line 115: "using AI to process clinical and genetic data" (not sure if this defintion is comprehensive enough as it highlights only AI here. Instead use the word 'technologies' instead of AI as it might be a generic representation.

Line 130: It might be helpful to mention the exact date of search instead of August 2025. If anyone wants to do another search the date will be helpful to know.

Line 139: Not sure if we are assessing risk of bias here in this review. Please modify accordingly.

Line 141: independently reviewed (does it include both title and abstract screening, and full text screening?) thought it might be good to clarify.

Line 143: consulting the research team (it might be helpful to provide more clarity here on who resolved the discrepancies)

Results:

Please verify the number of articles 4857 - the numbers do not seem to add up.

Table 1: Consistent use of terminology: eg., SMS and SMSs. Also, please provide abbreviation for SMS

Line 197: " while pharmacists, psychiatric technicians and primary care practitioners" is preferably. suggest you to remove the 'and'

Line 200: Consistent use of percentage will be helpful. Eight studies are provided with the % and missing in the first line with seven studies.

Line 207: suggestion: "mHealth was the commonly used digital technology in the included studies"

Line 207: Please rephrase the line with two dashes " - which described the use of mHealth - "

Line 208: Not sure what the word 'this' refers here.

Line 348: 'previous scoping review' instead of previous systematic review.

Conclusion: Might be good to mention that there are cost issues are there as well. Would be interesting to note if any of the studies included made any mention of the costs involved with the technology and implementation.

Reviewer #2: I would like to thank the authors, who integrated all of my suggestions in their article, which in my opinion is now ready to be published.

**********

7. PLOS authors have the option to publish the peer review history of their article (what does this mean?). If published, this will include your full peer review and any attached files.

**Do you want your identity to be public for this peer review?**  If you choose “no”, your identity will remain anonymous but your review may still be made public.

For information about this choice, including consent withdrawal, please see our Privacy Policy.

Reviewer #1: No

Reviewer #2: No

**********

[NOTE: If reviewer comments were submitted as an attachment file, they will be attached to this email and accessible via the submission site. Please log into your account, locate the manuscript record, and check for the action link "View Attachments". If this link does not appear, there are no attachment files.]

Figure Resubmissions:

While revising your submission, we strongly recommend that you use PLOS’s NAAS tool (https://ngplosjournals.pagemajik.ai/artanalysis) to test your figure files. NAAS can convert your figure files to the TIFF file type and meet basic requirements (such as print size, resolution), or provide you with a report on issues that do not meet our requirements and that NAAS cannot fix.

After uploading your figures to PLOS’s NAAS tool - https://ngplosjournals.pagemajik.ai/artanalysis, NAAS will process the files provided and display the results in the "Uploaded Files" section of the page as the processing is complete. If the uploaded figures meet our requirements (or NAAS is able to fix the files to meet our requirements), the figure will be marked as "fixed" above. If NAAS is unable to fix the files, a red "failed" label will appear above. When NAAS has confirmed that the figure files meet our requirements, please download the file via the download option, and include these NAAS processed figure files when submitting your revised manuscript.

# Author comment: Author response to Decision Letter 2

**DOI:** 10.1371/journal.pgph.0006746.r004

13 Apr 2026

Attachment Submitted filename: Point by Point Response to reviewers_AGT.docx

# Peer review: Decision Letter 2

**DOI:** 10.1371/journal.pgph.0006746.r005

**Authors:** Zulkarnain Jaafar

5 May 2026

PGPH-D-25-02956R2

Use of Digital Health in Task-sharing for Prevention and Management of Non-Communicable Diseases in Africa: A Scoping Review

PLOS Global Public Health

Dear Dr. Mhlanga,

Thank you for submitting your manuscript to PLOS Global Public Health. After careful consideration, we feel that it has merit but does not fully meet PLOS Global Public Health’s publication criteria as it currently stands. Therefore, we invite you to submit a revised version of the manuscript that addresses the points raised during the review process.

==============================

EDITOR: Dear Author, please make necessary changes based on the comments provided by the reviewer.

==============================

Please submit your revised manuscript by 3rd June 2026. If you will need more time than this to complete your revisions, please reply to this message or contact the journal office at globalpubhealth@plos.org. When you're ready to submit your revision, log on to https://www.editorialmanager.com/pgph/ and select the 'Submissions Needing Revision' folder to locate your manuscript file.

Please include the following items when submitting your revised manuscript:

- A letter that responds to each point raised by the editor and reviewer(s). You should upload this letter as a separate file labeled 'Response to Reviewers'.
- A marked-up copy of your manuscript that highlights changes made to the original version. You should upload this as a separate file labeled 'Revised Manuscript with Track Changes'.
- An unmarked version of your revised paper without tracked changes. You should upload this as a separate file labeled 'Manuscript'.

Guidelines for resubmitting your figure files are available below the reviewer comments at the end of this letter.

As the corresponding author, your ORCID iD is verified in the submission system and will appear in the published article. PLOS supports the use of ORCID, and we encourage all coauthors to register for an ORCID iD and use it as well. Please encourage your coauthors to verify their ORCID iD within the submission system before final acceptance, as unverified ORCID iDs will not appear in the published article. *Only*  the individual author can complete the verification step; PLOS staff *cannot*  verify ORCID iDs on behalf of authors.

We look forward to receiving your revised manuscript.

Kind regards,

Zulkarnain Jaafar

Academic Editor

PLOS Global Public Health

Journal Requirements:

If the reviewer comments include a recommendation to cite specific previously published works, please review and evaluate these publications to determine whether they are relevant and should be cited. There is no requirement to cite these works unless the editor has indicated otherwise.

Please review your reference list to ensure that it is complete and correct. If you have cited papers that have been retracted, please include the rationale for doing so in the manuscript text, or remove these references and replace them with relevant current references. Any changes to the reference list should be mentioned in the rebuttal letter that accompanies your revised manuscript. If you need to cite a retracted article, indicate the article’s retracted status in the References list and also include a citation and full reference for the retraction notice.

Additional Editor Comments (if provided):

[Note: HTML markup is below. Please do not edit.]

Reviewers' comments:

Reviewer's Responses to Questions

**Comments to the Author**

1. If the authors have adequately addressed your comments raised in a previous round of review and you feel that this manuscript is now acceptable for publication, you may indicate that here to bypass the “Comments to the Author” section, enter your conflict of interest statement in the “Confidential to Editor” section, and submit your "Accept" recommendation.

Reviewer #1: All comments have been addressed

**********

2. Does this manuscript meet PLOS Global Public Health’s publication criteria? Is the manuscript technically sound, and do the data support the conclusions? The manuscript must describe methodologically and ethically rigorous research with conclusions that are appropriately drawn based on the data presented.

Reviewer #1: Yes

**********

3. Has the statistical analysis been performed appropriately and rigorously?

Reviewer #1: N/A

**********

4. Have the authors made all data underlying the findings in their manuscript fully available (please refer to the Data Availability Statement at the start of the manuscript PDF file)?

The PLOS Data policy requires authors to make all data underlying the findings described in their manuscript fully available without restriction, with rare exception. The data should be provided as part of the manuscript or its supporting information, or deposited to a public repository. For example, in addition to summary statistics, the data points behind means, medians and variance measures should be available. If there are restrictions on publicly sharing data—e.g. participant privacy or use of data from a third party—those must be specified.

Reviewer #1: Yes

**********

5. Is the manuscript presented in an intelligible fashion and written in standard English?

PLOS Global Public Health does not copyedit accepted manuscripts, so the language in submitted articles must be clear, correct, and unambiguous. Any typographical or grammatical errors should be corrected at revision, so please note any specific errors here.

Reviewer #1: Yes

**********

6. Review Comments to the Author

Please use the space provided to explain your answers to the questions above. You may also include additional comments for the author, including concerns about dual publication, research ethics, or publication ethics. (Please upload your review as an attachment if it exceeds 20,000 characters)

Reviewer #1: Dear authors,

Great job in addressing all the comments. I have only one comment related to the PRISMA flowchart. The numbers still do not add up. Records identified = 4857. Records removed = 3580. So, the number of records screened must be 1277 and NOT 1295. This is what I meant by the numbers not adding up.

**********

7. PLOS authors have the option to publish the peer review history of their article (what does this mean?). If published, this will include your full peer review and any attached files.

**Do you want your identity to be public for this peer review?**  If you choose “no”, your identity will remain anonymous but your review may still be made public.

For information about this choice, including consent withdrawal, please see our Privacy Policy.

Reviewer #1: No

**********

[NOTE: If reviewer comments were submitted as an attachment file, they will be attached to this email and accessible via the submission site. Please log into your account, locate the manuscript record, and check for the action link "View Attachments". If this link does not appear, there are no attachment files.]

Figure Resubmissions:

While revising your submission, we strongly recommend that you use PLOS’s NAAS tool (https://ngplosjournals.pagemajik.ai/artanalysis) to test your figure files. NAAS can convert your figure files to the TIFF file type and meet basic requirements (such as print size, resolution), or provide you with a report on issues that do not meet our requirements and that NAAS cannot fix.

After uploading your figures to PLOS’s NAAS tool - https://ngplosjournals.pagemajik.ai/artanalysis, NAAS will process the files provided and display the results in the "Uploaded Files" section of the page as the processing is complete. If the uploaded figures meet our requirements (or NAAS is able to fix the files to meet our requirements), the figure will be marked as "fixed" above. If NAAS is unable to fix the files, a red "failed" label will appear above. When NAAS has confirmed that the figure files meet our requirements, please download the file via the download option, and include these NAAS processed figure files when submitting your revised manuscript.

# Author comment: Author response to Decision Letter 3

**DOI:** 10.1371/journal.pgph.0006746.r006

19 May 2026

Attachment Submitted filename: Point_by_Point_Response_to_reviewers_auresp_3.docx

# Editor report: Decision Letter 3

**DOI:** 10.1371/journal.pgph.0006746.r007

**Authors:** Zulkarnain Jaafar

26 May 2026

PGPH-D-25-02956R3

Use of Digital Health in Task-sharing for Prevention and Management of Non-Communicable Diseases in Africa: A Scoping Review

PLOS Global Public Health

Dear Dr. Mhlanga,

Thank you for submitting your manuscript to PLOS Global Public Health. After careful consideration, we feel that it has merit but does not fully meet PLOS Global Public Health’s publication criteria as it currently stands. Therefore, we invite you to submit a revised version of the manuscript that addresses the points raised during the review process.

==============================

EDITOR: Dear Author, please make necessary changes based on comments provided by the reviewer.

==============================

Please submit your revised manuscript by 24th June 2026. If you will need more time than this to complete your revisions, please reply to this message or contact the journal office at globalpubhealth@plos.org. When you're ready to submit your revision, log on to https://www.editorialmanager.com/pgph/ and select the 'Submissions Needing Revision' folder to locate your manuscript file.

Please include the following items when submitting your revised manuscript:

- A letter that responds to each point raised by the editor and reviewer(s). You should upload this letter as a separate file labeled 'Response to Reviewers'.
- A marked-up copy of your manuscript that highlights changes made to the original version. You should upload this as a separate file labeled 'Revised Manuscript with Track Changes'.
- An unmarked version of your revised paper without tracked changes. You should upload this as a separate file labeled 'Manuscript'.

Guidelines for resubmitting your figure files are available below the reviewer comments at the end of this letter.

As the corresponding author, your ORCID iD is verified in the submission system and will appear in the published article. PLOS supports the use of ORCID, and we encourage all coauthors to register for an ORCID iD and use it as well. Please encourage your coauthors to verify their ORCID iD within the submission system before final acceptance, as unverified ORCID iDs will not appear in the published article. *Only*  the individual author can complete the verification step; PLOS staff *cannot*  verify ORCID iDs on behalf of authors.

We look forward to receiving your revised manuscript.

Kind regards,

Zulkarnain Jaafar

Academic Editor

PLOS Global Public Health

Journal Requirements:

If the reviewer comments include a recommendation to cite specific previously published works, please review and evaluate these publications to determine whether they are relevant and should be cited. There is no requirement to cite these works unless the editor has indicated otherwise.

Please review your reference list to ensure that it is complete and correct. If you have cited papers that have been retracted, please include the rationale for doing so in the manuscript text, or remove these references and replace them with relevant current references. Any changes to the reference list should be mentioned in the rebuttal letter that accompanies your revised manuscript. If you need to cite a retracted article, indicate the article’s retracted status in the References list and also include a citation and full reference for the retraction notice.

Additional Editor Comments (if provided):

[Note: HTML markup is below. Please do not edit.]

Reviewers' comments:

[NOTE: If reviewer comments were submitted as an attachment file, they will be attached to this email and accessible via the submission site. Please log into your account, locate the manuscript record, and check for the action link "View Attachments". If this link does not appear, there are no attachment files.]

Figure Resubmissions:

While revising your submission, we strongly recommend that you use PLOS’s NAAS tool (https://ngplosjournals.pagemajik.ai/artanalysis) to test your figure files. NAAS can convert your figure files to the TIFF file type and meet basic requirements (such as print size, resolution), or provide you with a report on issues that do not meet our requirements and that NAAS cannot fix.

After uploading your figures to PLOS’s NAAS tool - https://ngplosjournals.pagemajik.ai/artanalysis, NAAS will process the files provided and display the results in the "Uploaded Files" section of the page as the processing is complete. If the uploaded figures meet our requirements (or NAAS is able to fix the files to meet our requirements), the figure will be marked as "fixed" above. If NAAS is unable to fix the files, a red "failed" label will appear above. When NAAS has confirmed that the figure files meet our requirements, please download the file via the download option, and include these NAAS processed figure files when submitting your revised manuscript.

# Author comment: Author response to Decision Letter 4

**DOI:** 10.1371/journal.pgph.0006746.r008

5 Jun 2026

Attachment Submitted filename: Point_by_Point_Response_to_reviewers_auresp_4.docx

# Editor report: Decision Letter 4

**DOI:** 10.1371/journal.pgph.0006746.r009

**Authors:** Zulkarnain Jaafar

10 Jun 2026

Use of Digital Health in Task-sharing for Prevention and Management of Non-Communicable Diseases in Africa: A Scoping Review

PGPH-D-25-02956R4

Dear Mhlanga,

We are pleased to inform you that your manuscript 'Use of Digital Health in Task-sharing for Prevention and Management of Non-Communicable Diseases in Africa: A Scoping Review' has been provisionally accepted for publication in PLOS Global Public Health.

Before your manuscript can be formally accepted you will need to complete some formatting changes, which you will receive in a follow up email. A member of our team will be in touch with a set of requests.

Please note that your manuscript will not be scheduled for publication until you have made the required changes, so a swift response is appreciated.

IMPORTANT: The editorial review process is now complete. PLOS will only permit corrections to spelling, formatting or significant scientific errors from this point onwards. Requests for major changes, or any which affect the scientific understanding of your work, will cause delays to the publication date of your manuscript.

If your institution or institutions have a press office, please notify them about your upcoming paper to help maximize its impact. If they'll be preparing press materials, please inform our press team as soon as possible -- no later than 48 hours after receiving the formal acceptance. Your manuscript will remain under strict press embargo until 2 pm Eastern Time on the date of publication. For more information, please contact globalpubhealth@plos.org.

Thank you again for supporting Open Access publishing; we are looking forward to publishing your work in PLOS Global Public Health.

Best regards,

Zulkarnain Jaafar

Academic Editor

PLOS Global Public Health

***********************************************************

Reviewer Comments (if any, and for reference):
